# Fatty acid DSF binds and allosterically activates histidine kinase RpfC of phytopathogenic bacterium *Xanthomonas campestris* pv. *campestris* to regulate quorum-sensing and virulence

**DOI:** 10.1371/journal.ppat.1006304

**Published:** 2017-04-03

**Authors:** Zhen Cai, Zhi-Hui Yuan, Huan Zhang, Yue Pan, Yao Wu, Xiu-Qi Tian, Fang-Fang Wang, Li Wang, Wei Qian

**Affiliations:** 1 State Key Laboratory of Plant Genomics, Institute of Microbiology, Chinese Academy of Sciences, Beijing, China; 2 School of Biological Sciences, University of Chinese Academy of Sciences, Beijing, China; University of Illinois, UNITED STATES

## Abstract

As well as their importance to nutrition, fatty acids (FA) represent a unique group of quorum sensing chemicals that modulate the behavior of bacterial population in virulence. However, the way in which full-length, membrane-bound receptors biochemically detect FA remains unclear. Here, we provide genetic, enzymological and biophysical evidences to demonstrate that in the phytopathogenic bacterium *Xanthomonas campestris* pv. *campestris*, a medium-chain FA diffusible signal factor (DSF) binds directly to the N-terminal, 22 amino acid-length sensor region of a receptor histidine kinase (HK), RpfC. The binding event remarkably activates RpfC autokinase activity by causing an allosteric change associated with the dimerization and histidine phosphotransfer (DHp) and catalytic ATP-binding (CA) domains. Six residues were found essential for sensing DSF, especially those located in the region adjoining to the inner membrane of cells. Disrupting direct DSF-RpfC interaction caused deficiency in bacterial virulence and biofilm development. In addition, two amino acids within the juxtamembrane domain of RpfC, Leu^172^ and Ala^178^, are involved in the autoinhibition of the RpfC kinase activity. Replacements of them caused constitutive activation of RpfC-mediated signaling regardless of DSF stimulation. Therefore, our results revealed a biochemical mechanism whereby FA activates bacterial HK in an allosteric manner, which will assist in future studies on the specificity of FA-HK recognition during bacterial virulence regulation and cell-cell communication.

## Author summary

Besides roles in nutrition, lipids also function as important signals in the regulation of prokaryotic and eukaryotic cells. In bacteria, fatty acids are part of the language of cell-cell communication known as quorum sensing for a decade. However, how bacteria detect these signals and regulate virulence remains elusive. Here, we provide multiple evidences to show that a full-length receptor histidine kinase, RpfC, directly binds to a fatty acid-based signal factor using a short sensor region. This binding event stimulates RpfC autokinase activity by triggering conformational change in its catalytic region, which is critical in regulating bacterial quorum sensing and virulence. Our results confirm a long-outstanding assumption in cell signaling of phytobacteria, and provide a technical pipeline to analyze fatty acid-receptor interactions.

## Introduction

Quorum-sensing is a process that bacterial cells communicate with each other to elicit specific physiological responses, including virulence against hosts [1,2]. How single-celled bacteria detect and respond to population density is a fundamental question in studying quorum sensing. Previous studies have reported that a number of chemicals, such as acylated homoserine lactones, peptides, quinolones, and small molecular fatty acids (FA), were implicated in the “bacterial languages” that are usually recognized by bacterial sensor histidine kinases (HK) to elicit quorum sensing [1,3]. Typically, HK and its cognate response regulator (RR) constitute a two-component signal transduction system (TCS), the predominant detection-response mechanism in prokaryotic cells. The N-terminal input region of an HK detects specific stimuli, and an invariant histidine residue within its C-terminal dimerization and histidine phosphotransfer (DHp) domain is autophosphorylated. The HK then modulates the phosphorylation level of the cytoplasmic RR by its phosphotransferase or phosphatase activity. Eventually, the RR uses its C-terminal output domain to regulate gene expression or cellular behavior [4]. In the past three decades, the basic biochemical processes of protein phosphorylation and dephosphorylation during TCS regulation have been well documented, however, as the first event to trigger the cell-cell communication, only a few of ligand-HK interactions were experimentally investigated [5,6]. Therefore, how HK recognizes various signals, especially in non-model bacteria, remains incompletely studied [7].

The major difficulty in studying HK-ligand interactions is the fact that the majority of HK are membrane-bound proteins with various hydrophobic helices [8]. Usually HK are enzymatic inactive in solutions containing detergents. Traditional strategies that express soluble truncated HK by deleting input regions and transmembrane helices then prevent the investigation of ligand-HK interactions [9,10]. In addition, HK are quite difficult to be crystalized so that the high resolution, three-dimensional structure of full-length HK is usually unavailable. This impedes the understanding of the structural mechanism of ligand-HK interaction [5]. Furthermore, signals like FA are hydrophobic molecules whose dissolution require organic solvents, these properties made the HK-ligand interactions difficult to be measured by commonly used biophysical methods, such as surface plasmon resonance (SPR) and isothermal titration calorimetry (ITC) [11,12]. Therefore, multi-disciplinary approaches based on extensive genetic analysis are needed to investigate FA-HK relationships.

In bacteria, diffusible signal factor (DSF) is a special family of signaling FA molecules. Various DSF-family members (such as DSF, BDSF, CDSF, SDSF, etc.) have been found to control quorum sensing and virulence in a number of bacteria, including plant pathogen *Xanthomonas* spp. and human pathogens *Pseudomonas aeruginosa*, *Stenotrophomonas maltophilia*, and *Burkholderia cepacia* [13,14]. These FAs take part in inter-species and inter-kingdom communications between bacteria and other organisms, including bacteria, fungi and host plants [15–21]. The first identified molecule of this family, DSF, was found in the Gram-negative bacterium *Xanthomonas campestris* pathovar (pv.) *campestris*, causal agent of black rot disease of cruciferous plants which encode approximately 52 HKs [22]. DSF is a medium-chain FA with a *cis*-11-methyl-dodecenoic acid structure [23]. Previous studies have revealed that extracellular DSF stimulates a TCS, RpfC-RpfG, to control bacterial virulence and quorum-sensing [24,25]. Of them, RpfC encodes a putative hybrid-type HK with multiple phosphorylation sites, while its cognate RR, RpfG, was the first HD-GYP domain-containing protein proved to have the phosphodiesterase activity to hydrolyze second messenger c-di-GMP into GMP [26]. At low bacterial cell density, RpfC binds to and represses the DSF synthase RpfF by a receiver (REC) domain, preventing production of DSF [27]. At high cell density, high concentration of extracellular DSF activates RpfC-RpfG system to degrade c-di-GMP, releasing the suppression of c-di-GMP on a global transcription factor Clp that controls the expression of multiple virulence factors [15,28,29]. However, because of the afore-mentioned technical difficulties in studying membrane-bound HK-FA interactions, whether DSF is the ligand of RpfC and biochemical mechanism of RpfC activation is unknown. Recently, two PAS-domain-containing HKs, RpfS and RpfR, were shown to bind DSF in *X*. *campestris* and BDSF in *Burkholderia cepacia*, respectively [30,31]. However, both RpfS and RpfR are soluble, cytoplasmic proteins without transmembrane helices, they are probably cytoplasmic receptors of intracellular DSF and unlikely cell-surface receptors to sense extracytoplasmic stimuli. Therefore, how the bacterial pathogen detects DSF dispersed in external environment and triggers cell-cell communication remains an opening question.

In this work, we show that DSF directly binds to a short N-terminal sensor of RpfC, elevating levels of RpfC autophosphorylation. A group of amino acid residues within the region adjoining to membrane were indispensable to DSF-RpfC binding and autokinase activation. DSF-RpfC interaction resulted in allosteric change in the DHp and catalytic ATP-binding (CA) regions, which may promote *in trans* phosphorylation of RpfC. In addition, substitutions of two amino acids within the juxtamembrane domain of RpfC caused constitutive activation of the HK. Our data revealed the biochemical mechanism responsible for the interaction between HK and FA, and provided insight into bacterial signaling during cell-cell communication.

## Results

### DSF activates the autokinase activity of membrane bound RpfC

**Fig 1:**
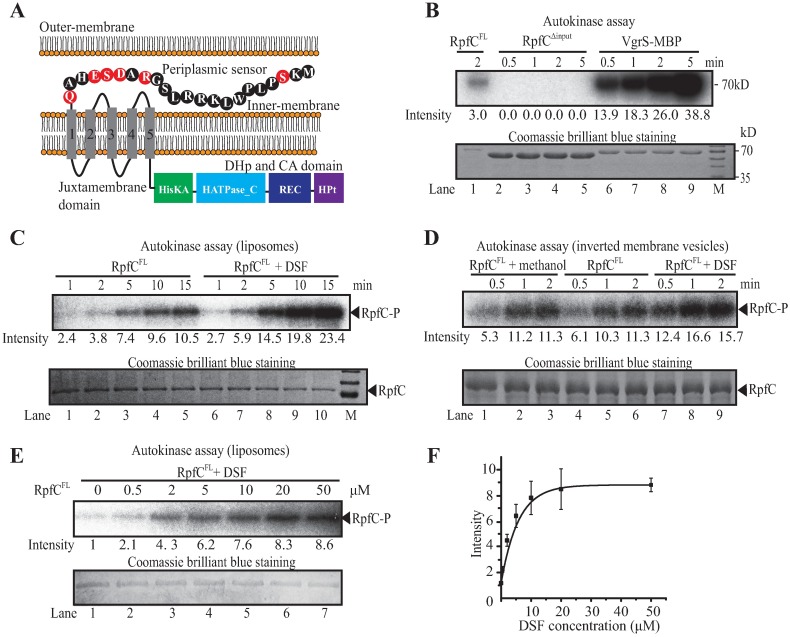
DSF stimulates the autokinase activity of full-length RpfC. (A) Schematic view of the secondary structure of full-length RpfC. The 22 amino acid residues of the N-terminal that are putatively located within the periplasmic space are depicted. Grey quadrangles (numbered 1–5) within the inner-membrane represent transmembrane helices. Domain names are according to the pfam database. (B) Truncated RpfC without input domain did not show autokinase activity. RpfC inverted membrane vesicles (IMV) (lane 1, 1 μg) and truncated histidine kinase VgrS (soluble) fused with a MBP (maltose binding protein, lanes 6–9, 10.7 μM for each) were used as positive controls. Lanes 2–5, samples containing 25.0 μM soluble RpfC^Δinput^. (C) DSF-stimulated autokinase activity of RpfC liposome. Lanes 6–10, 10 μM DSF was added together with ATP to the reaction mixture. The lower panels show proteins stained with Coomassie brilliant blue, which served as loading controls. (D) Full-length RpfC embedded in IMV exhibited autokinase activity that could be stimulated by DSF. All lanes, samples containing 1.4 μg RpfC^FL^ IMV. Lanes 7–9, 10 μM DSF was added to the reaction mixture of each sample together with ATP. (E-F) Quantification of the dose-dependent DSF stimulation of RpfC autokinase activity. (E) Autokinase activity of RpfC stimulated by different concentrations of DSF; (F) Quantification the band intensity of (E). Black bars represent the standard deviation (*n* = 3). Data points were fitted using a logarithmic model. Intensities of the autophosphorylation bands were estimated using Quantity One software. (B-E) Upper panels show the results of autokinase assays. Each lane contains 1.4 μg RpfC^FL^ liposome or 1.4 μg RpfC^FL^ IMV that was co-incubated with 100 μM ATP, including 10 μCi [γ-^32^P]-ATP, for indicated times. All reactions were immediately stopped and separated by 12% SDS-PAGE prior to autoradiography. Each experiment was independently repeated three times, and a representative experiment is shown.

RpfC belongs to a group of hybrid-type of HK with sensing mechanisms associated with membrane-spanning helices [8]. The putative secondary structure of RpfC has two characteristics different from the prototypical HKs (Fig 1A): Firstly, the signal input region of RpfC contains five hydrophobic TM helices and a putative 22-amino acid (aa)-length, periplasmic sensor at the most front end of its N-terminus. Secondly, there is a short juxtamembrane domain (16 aa-length), rather than a HAMP linker (about 50 aa-length), connects the input region to DHp-CA domains. In addition, RpfC also contains a C-terminal histidine phosphotransfer (HPt) domain and a REC domain (Fig 1A). The enzymatic activity of RpfC has never been investigated before. To biochemically confirm that RpfC is a HK, a truncated, soluble RpfC protein (RpfC^Δinput^) lacking the N-terminal input region (including sensor and TM domains) was obtained and purified. However, RpfC^Δinput^ did not exhibit any detectable autokinase activity (Fig 1B), suggesting that the input region is critical for maintaining enzymatic activity. To address this question, we obtained a full-length RpfC protein (RpfC^FL^) with a C-terminal His_6_ epitope tag. Two membrane-embedded forms of RpfC^FL^, liposome and inverted membrane vesicle (IMV), were reconstructed and purified. As shown in Fig 1C and 1D, both forms of RpfC^FL^ exhibited clear autokinase activity, making it possible to enzymatically investigate the mechanism of RpfC activation.

To determine if DSF affects the enzymatic activity of RpfC, DSF was added to reaction mixtures containing the liposome or IMV forms of RpfC^FL^. As shown in Fig 1C and 1D, the level of RpfC^FL^-P phosphorylation approximately doubled compared with the control. Kinetic analyses of the IMV and liposome forms of RpfC^FL^ showed an increase in the phosphorylation level of the IMV form at 30 s post DSF addition, whereas a similar increase was not detected until 2 min for the liposome form. This difference might be caused by variation in the phospholipid compositions of the IMV and liposome forms, which would affect autokinase activity. In addition, dose-response analysis of DSF on the activity of RpfC revealed that addition of 0.5 μM DSF was sufficient to elicit a detectable increase in the level of RpfC^FL^-P (Fig 1E and 1F). This concentration agrees with the previously reported minimal bioactive concentration of DSF (approximately 0.5 μM) that required for eliciting cell-cell communication [23]. Increasing the DSF concentration resulted in a logarithmic increase in the RpC^FL^-P level, and RpfC^FL^-P levels tapered off as they neared 20 μM (Fig 1F), suggesting that the system had reached saturation point.

RpfC is a hybrid histidine kinase that contains additional HPt and REC domains (Fig 1A). To exclude the possibility that the elevation of RpfC^FL^-P levels was caused by a change in DSF-dependent phosphoryl transfers from the DHp domain to these domains, the conserved phosphorylation sites within the HPt and REC domains were independently replaced [RpfC^H657A^ and RpfC^D512V^]. The IMV forms of the two recombinant RpfC proteins were used in the phosphorylation assay. As shown in S1 Fig, neither of the amino acid replacement affected the DSF-dependent elevation of RpfC autokinase activity. Taken together, these findings provide direct biochemical evidences to demonstrate a long-term supposition that RpfC is an HK whose autokinase activity can be activated by the ligand DSF.

### The N-terminal input region of RpfC plays an essential role in DSF perception

**Fig 2:**
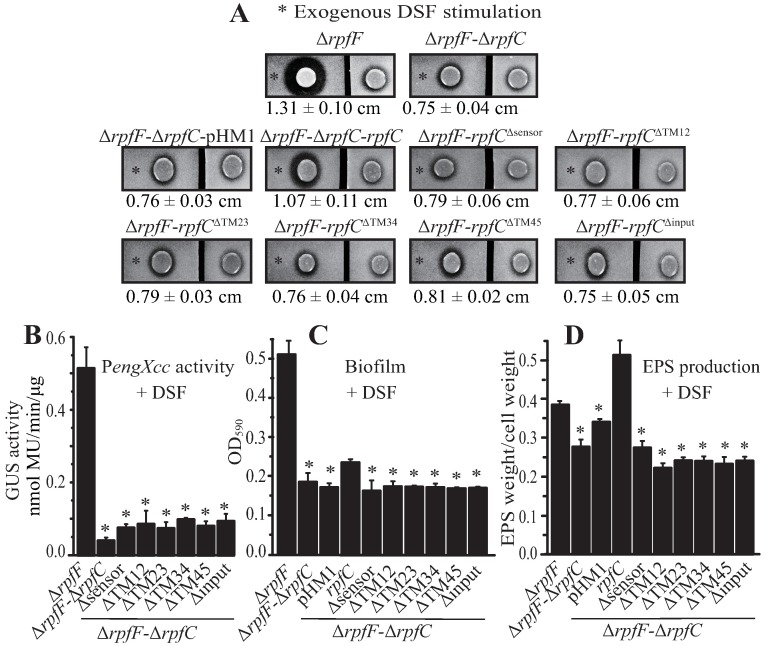
The input region of RpfC is essential in DSF sensing. (A) Putative sensor and transmembrane helices contribute to DSF detection. In each panel, duplicate bacterial colonies were inoculated on the left and right and were separated by cutting the NYG plate into two parts. A total of 3.5 μL of DSF (30 μM, indicated by an asterisk) were spotted near the colony on the left. The ability to produce extracellular proteases was observed after 36 h of incubation. Average diameters (cm) of the protein degradation zones of the left colony with standard deviations (*n* = 4) are listed below each panel. (B, C, and D) Mutation of the input region of *rpfC* compromised the ability of the bacteria to detect DSF and modulate the expression of *engXcc* (B), form a biofilm (C), or produce EPS (D). In (B), each bacterial strain contains a recombinant pHM2 vector with a P*engXcc*-*GUS* transcriptional fusion. In (B, C, and D), 10 μM exogenous DSF was added to bacterial cultures. GUS activities, EPS production, and biofilm development were measured after 9 h, 75 h and 12 h, respectively. Black bars represent standard deviation (*n* = 3). * = statistically significant (*P* ≤ 0.05) compared with the Δ*rpfF* strain, measured by the Student’s *t*-test.

Because membrane-bound HK usually employ periplasmic sensors and TM helices to detect signals, mutagenesis was used to identify regions critical for detecting DSF. A series of in-frame *rpfC* deletion mutants in a Δ*rpfF* genetic background that lost the capability to synthesize endogenous DSF were constructed. These constructs include a mutant with a deletion of the putative short sensor region (*rpfC*^Δsensor^), four mutants with their TM regions deleted in pairs to maintain the general topology of the protein (*rpfC*^ΔTM1-2^, *rpfC*^ΔTM2-3^, *rpfC*^ΔTM3-4^, *rpfC*^ΔTM4-5^), and a mutant with the input regions (sensor and TM regions) completely deleted (*rpfC*^Δinput^). Western blotting analyses revealed that deletions of the TM2–TM3 or input region caused instability of RpfC, whereas deletion of the short sensor region resulted in increase of the cellular levels of RpfC protein (S2A Fig), indicating the presence of a negative autoregulatory loop mediated by the sensor. As deletion of *rpfF* completely eliminated the synthesis of endogenous DSF, these double mutants were used to determine whether the different input regions were functional in sensing exogenously added DSF. Since the DSF-RpfC regulated, extracellular protease (EXP) activity can be directly observed without staining during bacterial growth [32], it was selected to be measured as a representative phenotype of the DSF-RpfC regulation. As compared with the positive control (Δ*rpfF*Δ*rpfC*-*rpfC*), EXP activities of all these mutants were severely decreased, most likely because they lost the ability to detect exogenous DSF stimulation (Fig 2A). To quantify the effect of DSF perception in these mutants, we used pHM2 vector to construct a biosensor (P*engXcc*-GUS) by fusing β-glucuronidase gene (*GUS*) to the promoter of *engXcc* (*XC_0639*), which encodes an extracellular endoglucanase specifically regulated by the RpfC-RpfG system [15,23]. After providing the biosensor *in trans* to these mutants, GUS activity assay revealed that the transcription level of *engXcc* was reduced to 13.9–18.6% of that of the control (Fig 2B). Similarly, when DSF was added to the bacterial cultures, the ability of these mutants to form biofilms and produce extracellular polysaccharides (EPS) was significantly decreased (Fig 2C and 2D), exhibiting deficiencies in sensing DSF.

**Fig 3:**
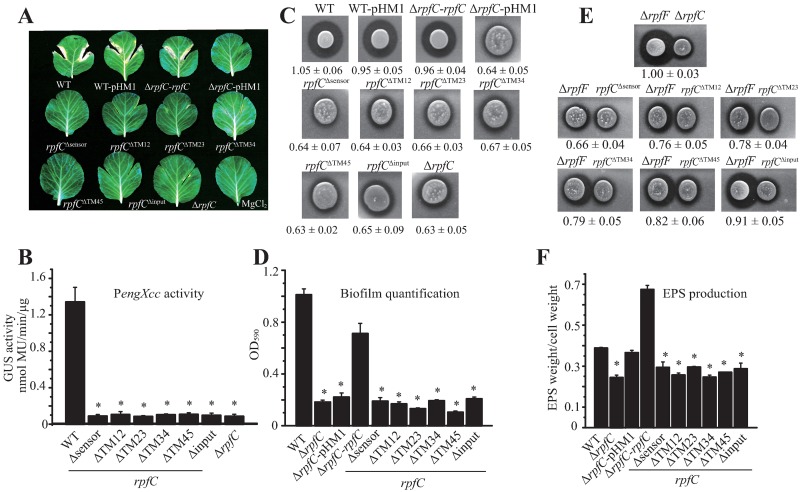
Putative N-terminal sensor and transmembrane helices are essential to RpfC regulation in virulence. (A) Mutations in the coding sequence of input region of *rpfC* decreased bacterial virulence. Bacterial strains were inoculated onto leaves of *Brassica oleraceae* cv. Jingfeng No. 1 by scissor cutting. Virulence levels were estimated 10 days post-inoculation. Quantification of the virulence scores was showed in S3A Fig. (B) Mutations in *rpfC* decreased expression of *engXcc*. Transcriptional activity of *engXcc* was measured by estimating the GUS activity. Each strain contained a recombinant pHM2 vector (pHM2::P*engXcc*-*GUS*). Black bars represent standard deviation (*n* = 3). (C) Mutations in *rpfC* decreased extracellular protease production. A 2-μL aliquot of each bacterial culture (OD_600_ = 0.4) was inoculated onto NYG-milk plates and incubated at 28°C for 36 h. Average diameters (cm) of protein degradation zones (*n* = 4) are shown in the figure. (D) Mutations in *rpfC* decreased biofilm production. Bacterial strains were cultured in 96-well polystyrene plates in NYG medium at 28°C for 12 h. Biofilm production was measured by crystal violet staining. Black bars represent standard deviation (*n* = 4). (E) Estimation of DSF production by recovery of extracellular protease production by the *rpfF* mutant. In each panel, the *rpfF* mutant (Δ*rpfF*, no DSF production) was inoculated on the left of the NYG-milk plate, and *rpfC* mutants were inoculated on the right. Strains were cultured at 28°C for 36 h. Diagram showing average protein degradation zones (cm, *n* = 3) of the Δ*rpfF* strain is shown below. (F) Mutations in *rpfC* decreased extracellular polysaccharide (EPS) production. In (B, D and F), * = statistically significant (*P* ≤ 0.05) compared with the WT strain (B) or Δ*rpfC*-*rpfC* (D and F), measured by the Student’s *t*-test.

To further investigate the regulatory function of the input region of RpfC in virulence, the same deletions as described above were generated in a wild-type (WT) background. Phenotypic characterization revealed that bacterial virulence (Fig 3A and S3A Fig), EXP production (Fig 3C), biofilm formation (Fig 3D), and EPS production (Fig 3F) of these mutants were all significantly reduced compared with the control. Quantification of the P*engXcc-GUS* activity (provided *in trans* by a recombinant pHM2 vector in each strain) showed that *engXcc* expression levels in these mutants decreased to 6.2–7.9% of the WT level (Fig 3B). In addition, as *rpfC* negatively modulates DSF synthesis, to measure the DSF production of the mutants, each strain was spotted onto a NYG-milk plate in close proximity to a Δ*rpfF* mutant deficient in endogenous DSF production. As shown in Fig 3E, deletion of the sensor region did not release the suppression of DSF production, while deletion of the transmembrane regions (TM12-TM45) moderately decreased the inhibition of DSF synthesis by RpfC, since the *rpfF* mutant exhibited higher EXP activity to degrade milk. Deletion of the entire input domain (sensor and TM regions) resulted extensive secretion of EXP, suggesting that the RpfC-mediated inhibition of DSF synthesis is remarkably eliminated.

Taken together, mutational analyses suggested that the sensor and TM regions of RpfC are all involved in DSF detection and regulation of bacterial virulence. Of these, the function of the RpfC sensor appears to be particularly important as its deletion resulted in an increase in the cellular amount of RpfC protein, and had no effect in suppression of DSF-regulated EXP production as the other regions of input domain. The following analysis therefore mainly focused on the possible interaction between the RpfC sensor and DSF.

### Identification of amino acids essential for sensing DSF

**Fig 4:**
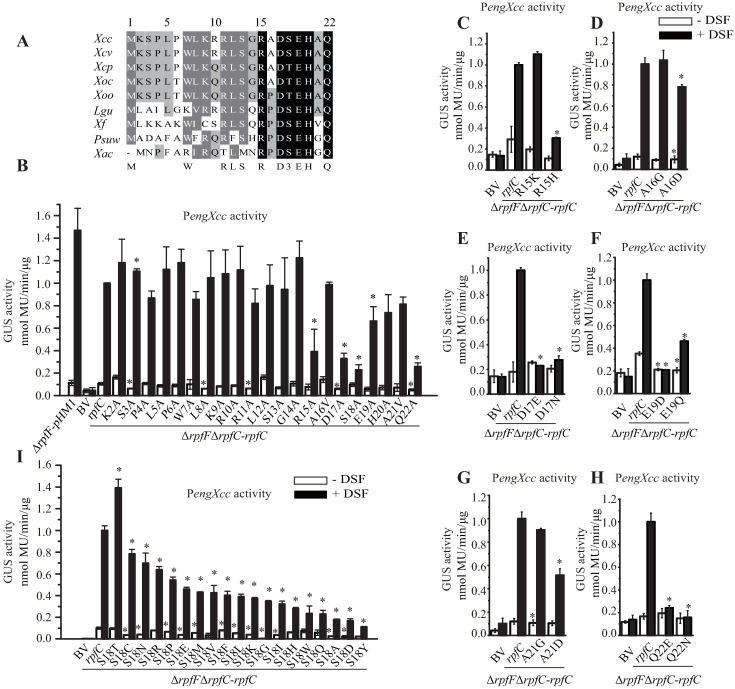
Identification of essential amino acids for detection of DSF. (A) Alignment of RpfC sensor sequences of orthologs from close-relatives of *X*. *campestris*. *Xcc*: *X*. *campestris* pv. *campestris*; *Xcv*: *X*. *campestris* pv. *vesicatoria*; *Xcp*: *X*. *campestris* pv. *pelargonii*; *Xoc*: *X*. *oryzae* pv. *oryzicola*; *Xoo*: *X*. *oryzae* pv. *oryzae*; *Lgu*: *Lysobacter gummosus*; *Xf*: *Xylella fastidiosa*; *Psuw*: *Pseudoxanthomonas suwonensis*; *Xac*: *X*. *axonopodis* pv. *citri*. All bacterial species belong to the Xanthomonadaceae. (B) Alanine-scanning mutagenesis of the RpfC sensor. *rpfC* containing a corresponding mutation was provided *in trans* in the Δ*rpfF*Δ*rpfC* double mutant on a pHM1 vector. The P*engXcc-GUS* sequence was fused in the bacterial chromosome. GUS activity was estimated in the absence (white frame) and presence (black frame) of DSF (10 μM). (C-H) The role of substitutions of identified essential amino acids in P*engXcc activity*. (I) Substitution of the Ser^18^ impacts the P*engXcc* activity. The Ser^18^ within RpfC sensor was replaced by other 19 amino acids. The experiments of (C-I) were performed as described in (B). In (B-I), standard deviation (*n* = 3) was estimated. * = statistically significant (*P* ≤ 0.05) compared with the control strain (Δ*rpfF*Δ*rpfC-rpfC*), measured by the Student’s *t*-test.

We proposed that the RpfC sensor contains the amino acids essential for sensing the DSF signal. Multiple-Alignment of the RpfC sensor sequences from orthologs of close-relative bacteria of *X*. *campestris* pv. *campestris* showed that 15^th^ to 22^nd^ amino acids are highly conserved in species belonging to the Xanthomonadaceae family (Fig 4A), indicating this region is critical in function. Thereafter, alanine-scanning mutagenesis was used to identify essential amino acids in recognizing DSF. A full-length *rpfC* sequence was first amplified and then inserted into the pHM1 vector. Next, 19 non-Ala codons, except the initial Met^1^ residue, within the sensor region were individually point mutated into the Ala codon. The two indigenous Ala (Ala^16^ and Ala^21^) were also individually replaced into Val (Fig 1A). These 21 recombinant vectors were then individually electroporated into a Δ*rpfF*Δ*rpfC* double mutant strain containing a *GUS* reporter fused to the promoter region of *engXcc* on the bacterial chromosome. Western blotting analysis revealed that apart from RpfC^K2A^, whose cellular amount was relatively low, all recombinant RpfC proteins were stable (S2B Fig). An exogenous DSF stimulation assay was then used to compare the *engXcc* transcription levels of these mutants to that of the positive control strain containing a plasmid-borne *rpfC*. As shown in Fig 4B, in the absence of exogenous DSF, replacements of S3A, L8A, R11A, D17A and Q22A caused slightly but significant changes in P*engXcc* activity (*P* ≤ 0.05). In the presence of exogenous DSF, three replacements, D17A, S18A, and Q22A, caused significant reductions in the P*engXcc* activity (levels 22.9–32.9% of the WT level). The R15A and E19A substitutions resulted in significant but intermediate reductions in the activation of the P*engXcc* (39.0–66.1% of the WT level). In contrast, the S3A replacement caused a stable, significantly increase in the P*engXcc* activity (to 110.0% of that of the control, Fig 4B).

The above result revealed that substitutions in the amino acids from Arg^15^ to Gln^22^ (except Ala^16^, His^20^ and Ala^21^) of RpfC sensor resulted in deficiencies in sensing DSF. Consequently, further amino acid substitution analyses were conducted, which includes construction of recombinant strains containing a plasmid-borne RpfC^R15K^, RpfC^R15H^ (Fig 4C), RpfC^D17E^, RpfC^D17N^ (Fig 4E), RpfC^S18T^, RpfC^S18C^ (Fig 4I), RpfC^E19D^, RpfC^E19Q^ (Fig 4F), RpfC^Q22E^, and RpfC^Q22N^ (Fig 4H) in the genetic background of Δ*rpfF*Δ*rpfC* double mutations. Under DSF stimulation, RpfC^R15K^ strain has similar P*engXcc* activity to that of the control (110% level) in sensing DSF (Fig 4C), suggesting that the positively charged, polar residues with similar side chains (R or K) are important in this location. RpfC^S18T^ substitution, which naturally occurred in several species belonging to the genus *Xanthomonas* (Fig 4A), significantly increased the P*engXcc* activity to the 139% level of the control (Fig 4I), implying that the hydroxyl oxygen in the side chain of Ser (S) or Thr (T) is essential to sense DSF. However, all the other substitutions resulted in significant decreases of the P*engXcc* activity, indicating that these amino acids in the RpfC sensor region are essential and evolutionarily fixed to these locations (Fig 4C–4I). In addition, recombinant strains containing RpfC^A16G^, RpfC^A16D^ (Fig 4D), RpfC^A21G^ and RpfC^A21D^ (Fig 4G) were also constructed. P*engXcc* activity assay showed that RpfC^A16G^ and RpfC^A21G^ replacements did not impact the DSF perception (Fig 4D and 4G). If the nonpolar Ala in the two sites were replaced by the negatively charged, polar Asp that disrupts the native conformation of the sensor region, the P*engXcc* activities were significantly decreased to 78% and 52% of the control, respectively (Fig 4D and 4G).

Since the RpfC^S18T^ substitution increased the P*engXcc* activity under DSF treatment and is the naturally occurred variation (Fig 4A and 4I), we further constructed 16 recombinant strains in the genetic background of Δ*rpfF*Δ*rpfC* double mutant. Each strain contains a replacement of Ser^18^ of RpfC to one of the other 16 amino acids besides afore-mentioned Ala, Thr, and Cys. Quantification of P*engXcc* activity showed that except the RpfC^S18T^ replacement, all the other substitutions led to significantly decrease in the *engXcc* expression (Fig 4I), supporting the view that a Ser/Thr residue in this site is critical in sensing DSF.

### Replacements of essential amino acids affect DSF perception and bacterial virulence

**Fig 5:**
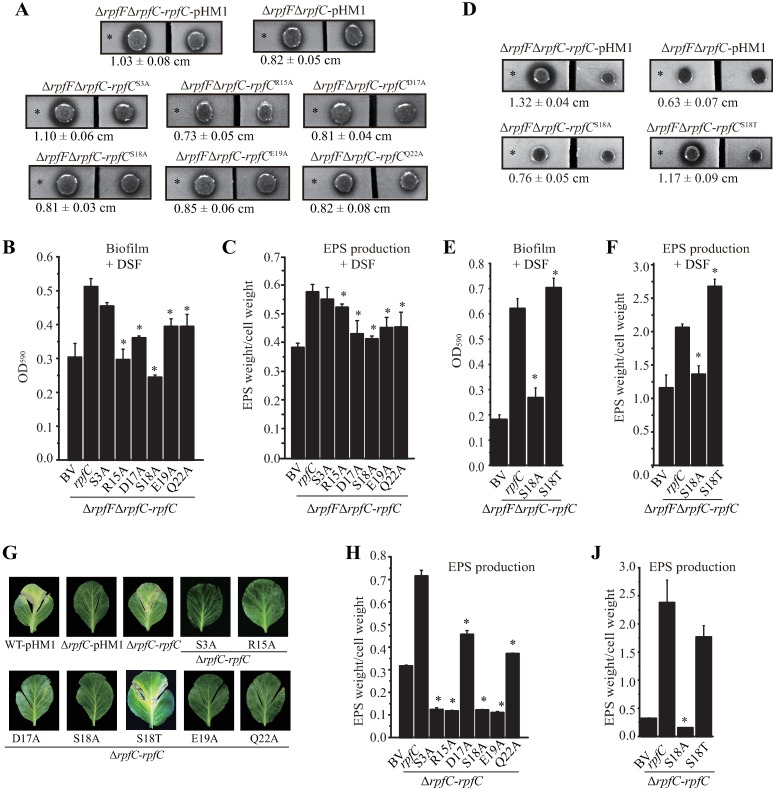
Impact of substitution of essential amino acids in RpfC sensor on DSF perception and bacterial virulence. (A and D). Amino acid substitution affects extracellular protease activity of bacteria. In each panel, bacterial colonies on the left and right are the same but were separated by cutting the NYG plate into two parts. A total of 3.5 μL of DSF (30 μM, indicated by an asterisk) were spotted near the left colony. Recovery of the ability to produce extracellular proteases was observed after 36 h of incubation. Average diameters (cm) of the protein degradation zones with standard deviations (*n* = 4) are listed below each panel. (B and E). Amino acid substitution affects biofilm formation of bacteria. Quantification of biofilm was performed by crystal violet staining methods. DSF was added to each bacterial culture at 10 μM prior to biofilm quantification. * indicates significant different to that of the control (Δ*rpfF*Δ*rpfC*-*rpfC*, *n* = 4), calculated by a Student’s *t*-test (*P* ≤ 0.05). (C, F, H and J). Amino acid substitution affects exopolysaccharides (EPS) formation of bacteria. Bacterial strains were cultured in NYG medium with 10 μM DSF for 72 hours (C, and H) or 96 hours (F and J) before EPS quantification. * indicates significant different to that of the control (Δ*rpfF*Δ*rpfC*-*rpfC*, *n* = 4), calculated by a Student’s *t*-test (*P* ≤ 0.05). (G) Bacterial virulence against host plant *B*. *oleraceae* cv. Jingfeng No. 1. Eight-week old plant was inoculated with bacterial cultures by sterile scissors. The levels of bacterial virulence were estimated 10 days after inoculation. A semi-quantification of virulence scale was shown in S3B and S3C Fig BV: Δ*rpfF*Δ*rpfC* strain containing a blank pHM1 vector.

To genetically evaluate the biological roles of the identified amino acids in sensing DSF, phenotypes of the recombinant bacterial strains (in the background of Δ*rpfF*Δ*rpfC* double mutant) were examined following addition of exogenous DSF. Compared with the *rpfC* complementation strain, the strain containing RpfC^S3A^ replacement showed similar levels of EPS production, biofilm formation and EXP production (Fig 5A–5C), while strains containing RpfC^R15A^, RpfC^D17A^, RpfC^S18A^, RpfC^E19A^, and RpfC^Q22A^ replacements exhibited significant decreases in EXP and EPS production, as well as in biofilm formation (Fig 5A–5C). Strains containing a RpfC^S18T^ substitution had similar or increased levels as the control in EXP activity, EPS production and biofilm formation (Fig 5D, 5E and 5F). Collectively, these genetic analyses suggest that five amino acids of Arg^15^, Asp^17^, Ser^18^, Glu^19^, and Gln^22^ adjoining to the transmembrane helices of RpfC are essential in detecting DSF, which is in parallel to the fact that they are highly conserved in bacterial evolution.

Besides DSF perception, the roles of these essential amino acids in controlling bacterial virulence were also analyzed. Recombinant bacterial strains were constructed in the Δ*rpfC* background, each contains a pHM1 vector to produce a RpfC derivate with an amino acid replacement. Western blotting analysis revealed that RpfC protein amounts of these strains were stable (S2J and S2K Fig). Plant inoculation assays showed that R15A, D17A, S18A, E19A and Q22A substitutions resulted in substantial attenuation in virulence against host cabbage *B*. *oleraceae* (Fig 5G and S3B Fig), while the S18T replacement did not affect bacterial virulence (Fig 5G and S3C Fig). In addition, the production of one of the major virulence factors of *X*. *campestris*, EPS, was also significantly decreased in all of the tested strains except of the one containing RpfC^S18T^ replacement (Fig 5H and 5J). These data strongly suggested that deficiency in DSF binding and perception resulted in deficiencies in bacterial virulence. It is noticeable that S3A substitution also resulted in remarkably decrease in virulence, albeit it caused slight hypersensitivity in detecting DSF as afore-mentioned (Fig 5G and S3B Fig). This result suggests that besides DSF perception, S3A is involved in additional, unknown function in regulating virulence.

### RpfC directly binds to DSF at the short sensor region

**Fig 6:**
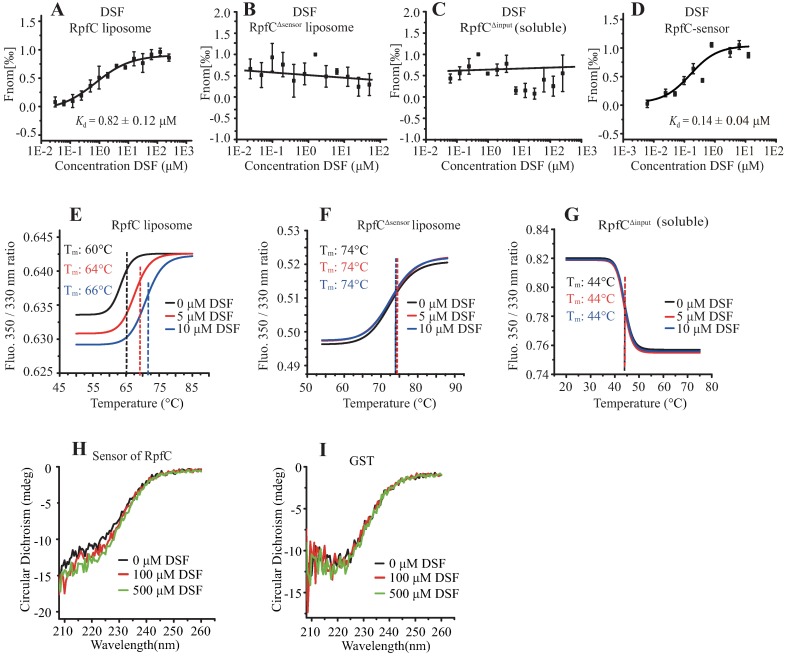
DSF directly binds to the sensor region of RpfC. (A) DSF binds to RpfC liposome. The concentrations of RpfC liposome were consistently measured as 0.1 μM. (B) RpfC liposome with sensor region deleted did not bind to DSF. (C) Cytosolic RpfC without input region did not bind to DSF. Soluble RpfC^Δinput^ (0.1 μM) was used in the assay. (D) DSF binds to a purified peptide of the RpfC-sensor. In (A-C), titrations of DSF ranged from 0.06 to 2000 μM. In (D), titrations of DSF ranged from 0.0122 to 25 μM. The solid curve is the fit of the data points to the standard KD-Fit function. Each binding assay was repeated independently three times, and black bars represent standard deviations. *K*_d_ = dissociation constant. (E-G) Representative melting curves of RpfC in temperature shift assay with differential scanning fluorimetry. Melting temperatures (T_m_) of the RpfC liposome, RpfC^Δsensor^ liposome, and soluble, RpfC^Δinput^ protein were shown as average of three independent measurement (*n* = 3). (H and I) Comparison of the CD spectra of RpfC sensor with various concentrations of DSF. The different concentrations of DSF are as indicated. The concentration of the purified RpfC sensor and GST were 60 μM and 6 μM, respectively. The measurements were carried out at room temperature. The data are representative of two independent experiments.

To detect a possible direct DSF-RpfC interaction, microscale thermophoresis (MST) was used because this technique is superior in studying membrane-bound receptors that are embedded in liposomes or nanodiscs with diverse ligands such as fatty acids and metals [33,34]. As shown in Fig 6A, DSF bound to the RpfC^FL^ liposome with a dissociation constant (*K*_d_) of 0.82 ± 0.12 μM, which represents a stronger binding affinity than those of the RpfS-DSF and RpfR-DSF interactions [31,35]. However, DSF didn’t bind to the truncated RpfC^Δsensor^ liposome (Fig 6B) and soluble RpfC protein without input region (RpfC^Δinput^, Fig 6C). Thermal shift assay (TSA) using differential scanning fluorimetry was also employed to measure the DSF-RpfC interaction. As shown in Fig 6E, during thermal denaturation, addition of DSF resulted in the significant increase of melting temperature (T_m_) of RpfC^FL^ liposome from 60.27°C to 64.27°C (5 μM DSF vs. 10 μM RpfC) or 66.27°C (10 μM DSF vs. 10 μM RpfC), strongly supporting a direct binding between DSF and RpfC. When the RpfC^Δsensor^ liposome and soluble RpfC^Δinput^ were applied in TSA, no thermal shift was detected after DSF stimulation (Fig 6F and 6G). These MST and TSA results suggest that DSF binds to the sensor region of RpfC. To directly detect the sensor-DSF interaction, the sensor peptide fused with a glutathione S-transferases (GST) tag was successfully obtained by a pGEX6P-1 expression system, and this sensor peptide was purified by on-column cleavage together with size exclusion chromatography to remove the GST tag. MST analysis revealed that DSF bound to the sensor peptide with a similar affinity of the full-length RpfC liposome (*K*_d_ = 0.14 ± 0.04 μM, Fig 6D). Since this sensor peptide only contains a tryptophan (Trp^7^), its autofluorescence is quite low and not applicable in TSA, circular dichroism spectra (CD) analysis was used to measure the effect of DSF stimulation on the secondary structure of sensor peptide. CD analysis showed that after addition of DSF, the secondary structure of the short peptide was remarkably changed. The ratio of strand was gradually elevated along with the increase of DSF concentration (Fig 6H), while DSF stimulation did not have recognizable impact on the secondary structure of GST (Fig 6I).

We further measured the impacts of substitutions of identified essential amino acids on the binding affinity with DSF. RpfC^FL^ with corresponding replacements of amino acids in the sensor region were purified, assembled into liposomes, and their interactions with DSF were measured by MST. As shown in S4 Fig, substitutions of RpfC^R15A^, RpfC^D17A^, RpfC^S18A^, RpfC^E19A^ and RpfC^Q22A^ completely eliminated the binding between DSF and RpfC liposomes. In contrast, the RpfC^S3A^ replacement decreased the *K*_d_ value to 0.613 ± 0.34 μM (S4A Fig), indicating that the RpfC^S3A^ substitution slightly enhanced the DSF-RpfC interaction. Collectively, the above results experimentally demonstrated that DSF directly binds to the sensor domain of HK RpfC.

### DSF stimulates allosteric change in activating RpfC autokinase

**Fig 7:**
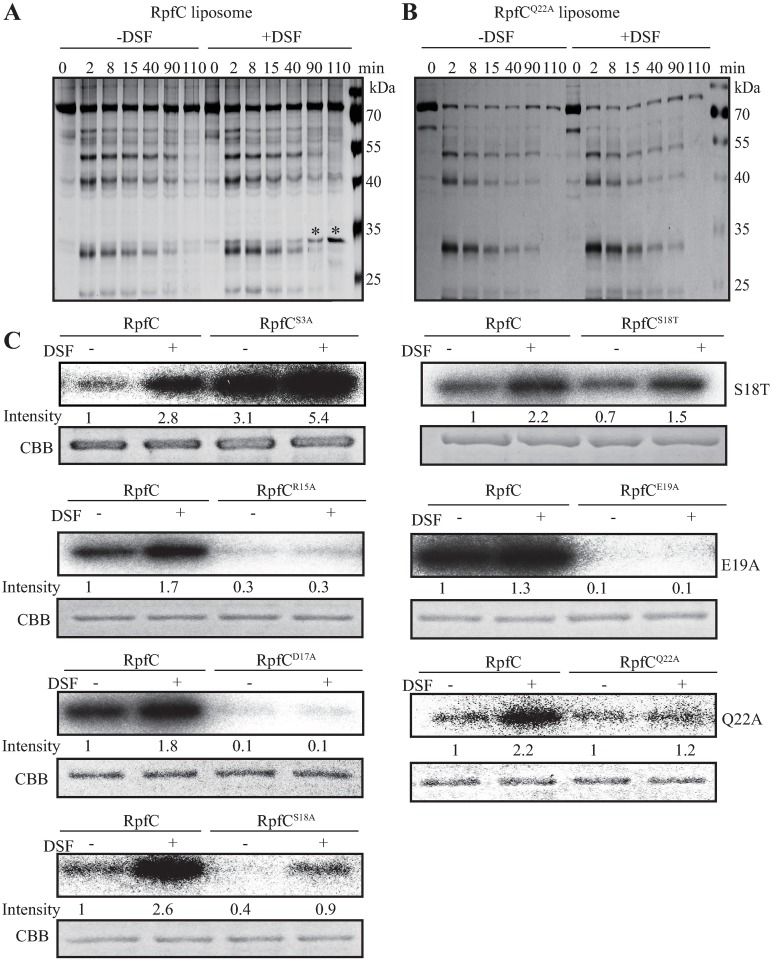
DSF stimulation changes the DHp-CA interface of RpfC. (A and B) Limited proteolysis of RpfC^FL^ liposomes. SDS-PAGE and molecular weight analyses of samples revealed DSF-dependent changes in trypsinolysis patterns, as indicated by asterisks. (A) Limited proteolysis of RpfC^FL^ liposome. (B) Limited proteolysis of RpfC^Q22A^ liposome. Limited proteolysis of other substitutions was shown in S5 Fig. (C) Replacement of essential residue affects RpfC^FL^ autokinase activity. Autokinase activities of RpfC liposomes containing corresponding amino acid replacement were measured by *in vitro* phosphorylation assay. DSF was added to reactions at 0.75 μM. Each lane contained 1.4 μg liposome phosphorylated by 100 μM ATP, including 10 μCi [γ-^32^P]-ATP, for 5 min. Experiments were independently repeated three times, and a representative experiment is shown.

Since the structures of RpfC and its orthologs remain unclear, limited proteolysis together with shotgun mass spectrometry were used to assess the conformational changes of RpfC involved in detecting DSF. During the analysis, the non-hydrolyzable ATP analog adenosine 5′(β,γ-imido) triphosphate (AMP-PNP) was added as a mimic for nucleotide binding. As shown in Fig 7A and S5 Fig, following addition of DSF to the reaction mixture, the patterns and amounts of most of the degraded RpfC^FL^ liposome fragments were similar to those of the control (DSF minus). However, DSF stimulation repeatedly caused a large accumulation of a protein fragments (30 kDa, including those in the S3A substitution, S5A Fig). Nano-LC-MS/MS analysis revealed that the band represented the DHp-CA region of RpfC (from 192nd to 474th aa.). Proteolysis of RpfC^FL^ liposomes with D17A, S18A, E19A, and Q22A replacements revealed similar degradation footprints, regardless of the presence or absence of DSF (Fig 7B and S5 Fig). These results suggest that the binding of DSF to the RpfC sensor caused conformational changes in the HK associated with the DHp-CA region.

To investigate whether substitutions of the identified essential amino acids influence the ability of the RpfC autokinase to react to DSF, phosphorylation levels of the liposomes of the RpfC^FL^ derivatives were compared to that of the WT. As shown in Fig 7C, stimulation of the RpfC^S3A^ substitution using a physiological concentration of DSF (0.75 μM) resulted in hypersensitivity in detecting DSF, with the RpfC-P level remarkably increased under stimulation. RpfC with R15A, D17A, S18A, and E19A replacements exhibited substantially decreased autokinase activities, regardless of the absence or presence of DSF. The Q22A replacement did not affect RpfC autophosphorylation in the absence of DSF, but decreased DSF sensing, as shown by the significant decrease in RpfC-P levels under DSF treatment (Fig 7C). In addition, the autophosphorylation level of RpfC^S18T^ was increased under DSF stimulation, albeit that the increase was slightly lower than that of the WT RpfC (Fig 7C). These results suggest that the identified essential amino acids, especially those located in the region adjoining to membrane, play important roles in RpfC autophosphorylation and DSF perception.

### Mutations in coding sequence of juxtamembrane domain suppress deficiency in sensing DSF

**Fig 8:**
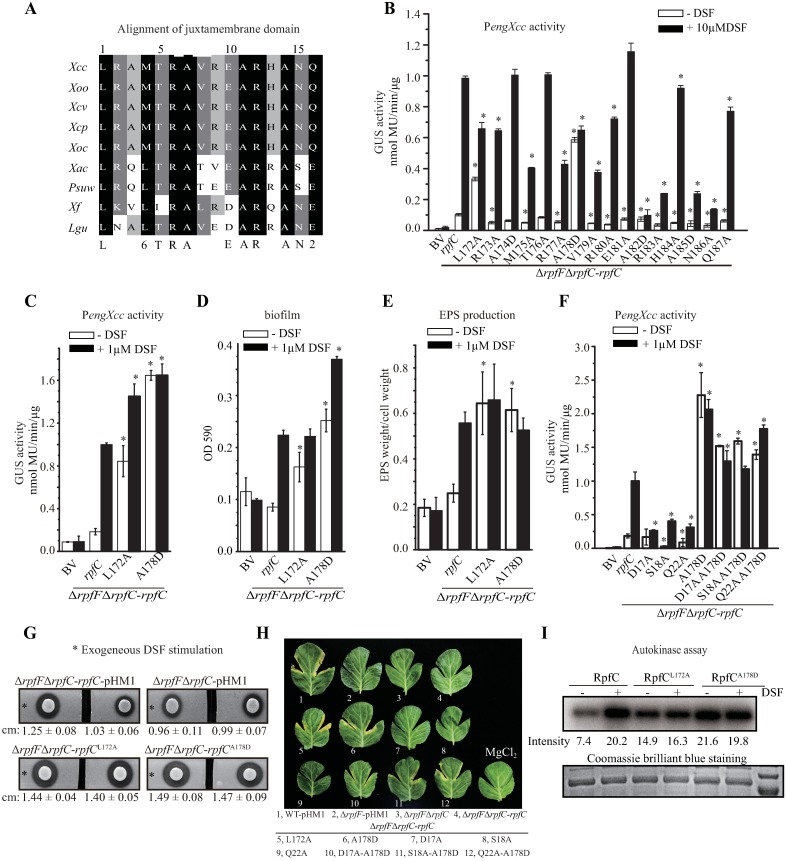
Leu^172^ and Ala^178^ in the juxtamembrane domain inhibit RpfC autokinase activity in responding to DSF stimulation. (A) Alignment of RpfC juxtamembrane domain sequences of orthologs from close-relative species of *X*. *campestris*. *Xcc*: *X*. *campestris* pv. *campestris*; *Xcv*: *X*. *campestris* pv. *vesicatoria*; *Xcp*: *X*. *campestris* pv. *pelargonii*; *Xoc*: *X*. *oryzae* pv. *oryzicola*; *Xoo*: *X*. *oryzae* pv. *oryzae*; *Lgu*: *Lysobacter gummosus*; *Xf*: *Xylella fastidiosa*; *Psuw*: *Pseudoxanthomonas suwonensis*; *Xac*: *X*. *axonopodis* pv. *citri*. (B, C and F) Mutational analyses of the *rpfC* juxtamembrane domain coding region. *rpfC* containing a point mutation was provided *in trans* in the Δ*rpfF*Δ*rpfC* double mutant on a pHM1 vector. The P*engXcc-GUS* sequence was fused in the bacterial chromosome. In (B), near-saturated concentration of DSF was added (10 μM). In (C and F), 1 μM DSF was added. (D and E) Impact of substitution in the juxtamembrane domain in biofilm formation and exopolysaccharide (EPS) production of bacteria. (D) Quantification of biofilm by crystal violet staining method. (E) Quantification of EPS production of bacteria. (F) Substitution of RpfC^A178D^ suppresses deficiency in DSF perception that is caused by mutations within sensor region. The P*engXcc-GUS* activity was measured and compared to that of the control. In (B-F) standard deviation (*n* = 3) was estimated. * = statistically significant (*P* ≤ 0.05) compared with the control strain (Δ*rpfF*Δ*rpfC-rpfC*), measured by the Student’s *t*-test. (G) RpfC^L172A^ and RpfC^A178D^ substitutions resulted in increase of extracellular protease (EXP) activity in the absence of DSF. In each panel, duplicate bacterial colonies were inoculated on the left and right and were separated by cutting the NYG plate into two parts. A total of 3.5 μL of DSF (10 μM, indicated by an asterisk) were spotted near the colony on the left. The ability to produce EXP was observed after 36 h of incubation. Average diameters (cm) of the protein degradation zones of the left colony with standard deviations (*n* = 3) are listed below each panel. (H) *rpfC*^L172A^ and *rpfC*^A178D^ mutations suppress deficiency in bacterial virulence caused by mutations in sensor region. Eight-week old host cabbage (*B*. *oleraceae* cv. Jingfeng No. 1) was used in bacterial inoculation. Virulence scores were recorded 10 days after inoculation. Quantification of virulence scores was showed in S3D Fig. (I) RpfC^L172A^ and RpfC^A178D^ proteins exhibited constitutively activated autophosphorylation. Each lane contains 1.4 μg liposome of RpfC derivates that was co-incubated with 100 μM ATP containing 10 μCi [γ-^32^P]-ATP. All reactions were immediately stopped and separated by 12% SDS-PAGE prior to autoradiography. Each experiment was independently repeated three times, and a representative experiment is shown.

RpfC contains a 16 aa-length, short juxtamembrane domain between the transmembrane helices and DHp-CA domains (Fig 1A). The juxtamembrane domain is highly conserved among RpfC orthologs from close-relatives of *X*. *campestris* pv. *campestris* (Fig 8A), implying that it has an important role in activating the RpfC autokinase after ligand perception. Alanine-scanning mutagenesis was again used to analyze the function of this region. As shown in Fig 8B, under stimulation of near-saturated concentration of DSF, 13 amino acid substitutions (in the background of Δ*rpfF*Δ*rpfC*-*rpfC* background) gave rise to significantly decrease of the capability to sense DSF that is quantified by P*engXcc* activities. In the absence of DSF, although 12 replacements also caused significant decrease in the background P*engXcc* activities, it is noticeable that two substitutions, RpfC^L172A^ and RpfC^A178D^, exhibited significant elevation of P*engXcc* activities (Fig 8B). When a low concentration of DSF (1 μM) was applied in treatment, both strains with RpfC^L172A^ or RpfC^A178D^ substitution also show constitutive activation of RpfC (Fig 8C). Especially, the P*engXcc* activity of the strain containing RpfC^A178D^ replacement in the absence of DSF treatment even increased to the similar level to that of the DSF stimulation (Fig 8C).

The phenotypes of the bacterial strains were also characterized. Without DSF stimulation, recombinant bacterial strains containing RpfC^L172A^ and RpfC^A178D^, which were constructed in the genetic background of Δ*rpfC*Δ*rpfF* double mutation, produced more biofilm, EPS, and EXP towards the levels of the positive control strain (Δ*rpfF*Δ*rpfC*-*rpfC*) under DSF stimulation (Fig 8D, 8E and 8G). For example, in the absence of DSF treatment, RpfC^L172A^ and RpfC^A178D^ replacements caused significantly increases of bacterial EPS production to 259% and 247% levels of that of the control strain, respectively, similar to the EPS amounts that were generated under DSF stimulation.

Since RpfC^L172A^ and RpfC^A178D^ substitutions constitutively activated the RpfC-regulated processes regardless of the presence or absence of DSF, we reasoned that mutations in the codons of Leu^172^ or Ala^178^ suppress the phenotypic deficiencies caused by mutations in the codons of essential amino acids of the sensor region in detecting DSF. To challenge this, we selected the Ala^178^ site for further genetic epistatic analysis. Three double mutants in the background of Δ*rpfF*Δ*rpfC*-*rpfC*^A178D^ were constructed by point mutating the codon of Asp^17^, Ser^18^, or Gln^22^. P*engXcc* activity assay revealed that all of these double mutations (*rpfC*^D17A-A178D^, *rpfC*^S18A-A178D^ and *rpfC*^Q22A-A178D^) significantly suppressed the deficiency in the *engXcc* expression that is caused by the point mutations in the codons of essential amino acids (Fig 8F), regardless of DSF stimulation. In addition, although both deletion of *rpfF* gene and point mutation of the codons of essential amino acids sensing DSF caused serious decrease in bacterial virulence, RpfC^A178D^ substitution suppressed the deficiency by recovering bacterial virulence level toward that of the positive control (Fig 8H and S3D Fig). The above genetic analysis suggests that the Leu^172^ and Ala^178^ are involved in autoinhibition of RpfC kinase activity without DSF stimulation. *In vitro* autokinase assay then revealed that the autophosphorylation levels of the recombinant RpfC^L172A^ and RpfC^A178D^ proteins are remarkably higher than that of the WT RpfC in the absence of DSF treatment, exhibiting a constitutive activating state (Fig 8I).

Collectively, these results support a view that the juxtamembrane domain of RpfC inhibits its autokinase activity when the concentration of DSF is low. However, DSF perception by the sensor region releases this inhibition to activate the RpfC autophosphorylation. During this process, Leu^172^ and Ala^178^ in the juxtamembrane domain play critical roles in inhibiting the RpfC activity.

## Discussion

**Fig 9:**
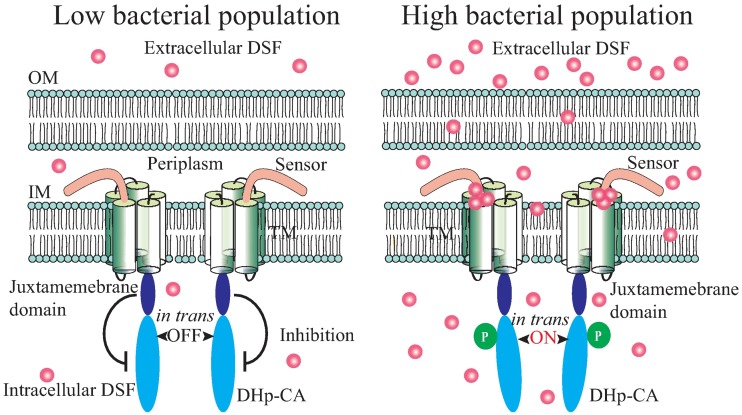
Molecular model of RpfC activation by DSF stimulation. Left panel: when bacterial population size and extracellular DSF concentration are low, RpfC autokinase activity is inhibited by its juxtamembrane domain (OFF state). Right panel: when bacterial population and extracellular DSF concentration are high, DSF binds to the RpfC sensor and releases the inhibition of the juxtamembrane domain on autokinase activity, resulting in RpfC autophosphorylation (ON state).

How a ligand interacts with HK is one of the fundamental questions in studying bacterial quorum sensing. Here, we provided enzymological, genetic and biophysical evidences to demonstrate that a HK of *X*. *campestris*, RpfC, is a *bona fide* membrane-bound receptor that directly binds a fatty acid signal, DSF. The results confirmed a long-held hypothesis regarding cell-cell communication in phytopathogenic bacteria [23,25,36]. DSF binds with high affinity to a 22-amino acid sensor region in the N-terminal of RpfC (Figs 1 and 6). The binding of DSF causes allosteric change associated with the DHp-CA domain of RpfC, which facilitates RpfC autophosphorylation (Fig 7). Systematic mutational investigation together with biochemical analysis identified six essential residues in the DSF-RpfC interaction (Figs 4 and 5). Of these, five amino acids located in the region adjoining to membrane are indispensable for DSF-RpfC binding (Figs 4 and 5), while the Ser^3^ of the RpfC sensor region is functionally unique, as replacement of this residue resulted in slight hypersensitivity in detecting DSF. In addition, two point mutations (*rpfC*^L172A^ and *rpfC*^A178D^) in the coding sequence of RpfC juxtamembrane domain effectively suppressed the phenotypic deficiencies caused by mutations in the sensor coding region, which is due to the constitutive activation of RpfC autokinase. These results support a molecular model (Fig 9) that the juxtamembrane domain inhibits the autokinase activity of RpfC when the extracellular concentration of DSF is low. However, when the DSF concentration increases along with the rise of bacterial population, DSF binds to the sensor region of RpfC and activates the HK by releasing this inhibition. To our best knowledge, this work provides the first experimental evidence to support a direct membrane-bound HK-FA interaction during bacterial quorum sensing and regulation of virulence.

In addition to their roles in nutrition, FA acts as important signaling molecules in both eukaryotes and prokaryotes. For example, in animals, free FA signals are detected by G-protein-coupled receptors (GPCR) or receptor tyrosine kinases (RTK) [37]. A recent structural study revealed that FA ligands of an human insulin secretion modulator, GPR40, bind to hydrophilic/positively charged residues in various docking sites that are formed by the characteristic seven-TM helices of GPCR [38]. In addition, complex FA, such as cholesterol, inhibits the autokinase activity of human RTK epidermal growth factor receptor (EGFR) by influencing membrane heterogeneity-mediated transmembrane signal transduction [39]. In the present work, MST and TSA analyses revealed that DSF binds RpfC liposome with a relatively strong affinity (*K*_d_ = 0.82 ± 0.12 μM, Fig 6A). This binding affinity is higher than those of the DSF-RpfR (1.37 μM, measured by ITC) and DSF-RpfS (1.40 μM, by ITC) interactions [31,35], while both RpfR and RpfS are cytosolic, soluble proteins without transmembrane helix. *In vitro* autokinase assay showed that 0.5 μM DSF was sufficient to activate RpfC autokinase (Fig 1). This concentration is very close to the reported minimum DSF concentration in eliciting cell-cell communication [23], and in the physiological range of extracellular DSF, which approximately ranged from 0.002 to 27.4 μM dependent on the growth stages of bacterial population [27,40,41].

Our data suggest that the N-terminal short sensor region of RpfC plays a fundamental role, if not exclusive, in directly binding DSF (Fig 6D and 6H). In the sensor region, five amino acids adjoining to membrane of the RpfC are highly conserved in the bacteria belonging to the Xanthomonadaceae family (Fig 4A). It is likely that these amino acids form a primary docking site for DSF. This hypothesis is supported by the following data: 1) Replacements of these residues completely dissociated the DSF-RpfC interaction (S4 Fig). 2) This region contains hydrophilic or charged residues, especially Ser^18^ which can be functionally substituted by a Thr residue and favors hydrogen bond formation, most possibly with the carboxyl group of DSF. 3) Replacement of these residues completely eliminated the DSF-triggered, conformational change associated with DHp-CA domain (Fig 7). 4) Amino acid replacements, especially of Ser^18^ and Gln^22^, decreased the level of autophosphorylation of RpfC in response to DSF (Fig 7). The above results suggest that the short sensor region of HK likely acts as a “hook” to catch DSF when it is diffusely passing through the periplasm. In a previous report, ITC analysis revealed that DSF binds to the PAS_4 domain of RpfS of *X*. *campestris* [31]. Collectively, it suggests that the binding sites of DSF to proteins are diverse and need to be investigated further.

It is noteworthy that the function of Ser^3^ within the RpfC sensor appears unique. This residue is highly conserved in all sequenced *Xanthomonas* species, whereas in other closely related bacterial species, Ala, Asp, Asn and Lys, respectively, occupy this position (Fig 4A). In the presence of exogenous DSF, S3A replacement caused hypersensitivity of RpfC in DSF detection: the binding affinity increased by approximately 20% compared with WT RpfC (S4 Fig), and the autokinase activity were also elevated (Fig 7C). However, as with the other essential residues, S3A replacement also attenuated virulence (Fig 5G and S3B Fig), suggesting that the Ser^3^ plays an additional role in regulating virulence, or DSF-RpfC binding affinity is subtly optimized during evolution so that any abnormality is detrimental to bacterial fitness. The role of Ser^3^ in binding DSF remains unclear. In previous study on the autoinducer CAI-I and HK CqsS in *Vibrio cholerae* [5], it revealed the length of hydrocarbon chain of autoinducer is critical in the ligand-HK interaction. The length of the FA chain is also a critical parameter in the activity of DSF-family compounds [23], therefore, one possible function of Ser^3^ is to act as a “ruler” to help suitable FA molecules gain entry into the RpfC docking site. However, further evidence is needed to clarify this hypothesis.

How a ligand activates a membrane-bound HK remains an important question. To date, several structural mechanisms for HK activation have been proposed [42], including scissor blade [43], piston-like change [44], or retortion triggered by ligand binding mechanisms [45]. In these models, the movement of CA domains is at the center of *in trans* phosphorylation of the homodimer of HK. Our data revealed that DSF stimulation resulted in conformational change associated with DHp-CA domain (Fig 7A). In addition, DSF-sensor interaction released the juxtamembrane domain-mediated autoinhibition on the RpfC kinase activity. Among them, Leu^172^ and Ala^178^ are two critical amino acids since replacement of them resulted in constitutive activation of RpfC. These results support a view that DSF acts as an allosteric activator of RpfC by releasing the autoinhibition of its juxtamembrane region. Further investigation is necessary to obtain the high resolution structure of the DSF-RpfC complex to dissect the structural mechanism of RpfC activation, which is also meaningful to study the specificity in sensing different DSF-family signals.

## Materials and methods

### Bacterial strains and plasmids

All bacterial strains and recombinant vectors used in this work are listed in S1 Table. *Xanthomonas campestris* pv. *campestris* (*Xcc*) 8004-derived strains and wildtype strain (WT) grew at 28°C in NYG medium (tryptone 5 g L^-1^, yeast extract 3 g L^-1^, glycerol 20 g L^-1^, pH 7.0) or 210 medium (sucrose 5 g L^-1^, casein enzymatic hydrolysate 8 g L^-1^, yeast extract 4 g L^-1^, K_2_HPO_4_ 3 g L^-1^, MgSO_4_·7H_2_O, 0.3 g L^-1^, pH 7.0). *E*. *coli* DH5α was used as the host for construction of all recombinant vectors. *E*. *coli* BL21(DE3) strain was used as the host for expressing recombinant proteins with pET30a vector (Novagen, USA). Appropriate antibiotics were added when needed as following concentrations: kanamycin (50 μg ml^-1^); spectinomycin (150 μg ml^-1^); ampicillin (100 μg ml^-1^) and rifamycin (25 μg ml^-1^). *Xcc* 8004 and *E*. *coli* electro-competent cells were prepared by extensively washing bacterial cells three times with ice-cold glycerol (10%). Transformation condition of both *X*. *campestris* pv. *campestris* and *E*. *coli* cells was set as 1.8 kV cm^-1^, 25 μF and 200 Ω and conducted in a Bio-Rad Pulser XCell (Bio-Rad, USA). HPLC purified diffusible signal factor (DSF, CAS No. 677354-23-3, purify > 90.0%) was purchased from Sigma Aldrich (USA) and used in different concentrations as indicated in different experiments.

### Construction of mutant and genetic complementation

If not specially mentioned, general molecular biology techniques, including PCR, DNA ligation, enzyme restriction, western blotting, etc, were according to the protocols in Molecular Cloning[46]. All in-frame deletion (markerless) mutants of *rpfC* and double mutant of *rpfC*-*rpfF* were constructed using suicide vector pK18mobsacB[47] by a homologous, double cross-over method. Briefly, the 5' and 3' genomic sequences of a targeted region were amplified using the primers listed in S2 Table, and correct PCR products were ligated into suicide vector pK18mobsacB. The recombinant pK18mobsacB vector was electroporated into competent cells of *Xcc* 8004 to generate single-crossover mutants by selection on NYG plates containing kanamycin. Afterwards, single-crossover mutants were cultured in NYG medium (antibiotic-free) for 1–2 hours and then grew on NYG plates containing 10% sucrose to select second-round homologous cross-overs. Correction of candidate bacterial mutants (resistant to 10% sucrose but sensitive to kanamycin) was verified by PCR and subsequent sequencing.

To genetically complement the Δ*rpfC* mutant, a full-length *rpfC* gene was amplified using primers listed in S2 Table, ligated into the broad-host vector pHM1 [48], and electroporated into *E*. *coli* DH5α to generate the recombinant vector. This vector was then extracted from *E*. *coli* DH5α and electroporated into the Δ*rpfC* or Δ*rpfC*Δ*rpfF* mutant, in which transcription of full-length *rpfC* was under the control of a P*lacZ* promoter.

### Alanine-scanning mutagenesis and construction of point mutations

Besides the initial residue Met^1^, the N-terminal sensor region of RpfC contains 21 residues, with two of them being Ala. To conduct alanine-scanning mutagenesis, full-length *rpfC* coding sequence was amplified by PCR and inserted into a pGEM T-easy vector (Promega, USA), and Easy Mutagenesis System (TransGen Biotech, China) was used to construct point mutations according to the manufactory’s manual. Coding sequences of 19 non-Ala residues were mutated into Ala, respectively, and Ala^16^ and Ala^21^ were mutated into Val, respectively. The point mutation was confirmed by sequencing. These inserts with corresponding point mutations were cut by restriction enzymes, purified, and ligated into broad host vector pHM1 [48]. Their expressions were under the control of a P*lacZ* promoter. Recombinant vectors were then electroporated into Δ*rpfC* or Δ*rpfC*Δ*rpfF* mutants as needed. Primers used to create these mutants are listed in S2 Table.

### GUS activity assay

To construct a biosensor using expression level of *engXcc*, which is subject to the control of RpfC-RpfG, as a parameter to estimate DSF-RpfC interaction, 5′ promoter region of *engXcc* (254 bp) was amplified by PCR, transcriptionally fused with a *gusE* gene to create P*engXcc*-GUS reporter insert (with native *gusE* Shine-Dalgarno to drive protein translation). For different purposes, the reporter sequence was provided *in trans* or integrated into bacterial chromosome. For *in trans* complementation, this reporter sequence was cloned into a pHM2 vector (no promoter upstream multiple cloning site), which was then electroporated into Δ*rpfC* or Δ*rpfC*Δ*rpfF* mutants for GUS expression analysis. For alanine-scanning mutagenesis, the P*engXcc*-GUS reporter sequence was integrated into the chromosomal locus of *engXcc* by homologous, double cross-over via a recombinant pK18mobsacB vector (pKengXcc-GUS).

For GUS activity assay, bacterial strains were cultured and adjusted to OD_600_ = 0.1, then grew without DSF or with 10 μM DSF for about 9 hours. Cells were collected by centrifugation (12,000 g, 10 min at 4°C), and immediately frozen in liquid nitrogen. GUS extraction buffer (50 mM sodium phosphate [pH 7.0], 5 mM DTT, 1 mM EDTA [pH 8.0]) was added to resuspend the cells and then these bacterial cells were lysed by sonication. The mixture was centrifuged (12,000 g, 10 min at 4°C) and the supernatant was used for GUS activity assay. Levels of GUS expression were quantified by its activity using 4-methylumbelliferyl ß-D-glucuronide (4-MUG, purchased from Sigma Aldrich, USA) as a substrate. A standard curve was prepared by diluting the 4-MU stock solution. The fluorescence of samples and standard curve solutions were measured using an excitation wave-length of 360 nm and an emission wave-length of 460 nm. Protein concentrations of supernatants were measured using Coomassie brilliant blue G-250 Protein Assay (Bio-Rad, USA) with BSA as a standard. For each experiment, at least three independent repeats were conducted for calculating the parameters.

### Bacterial virulence and phenotypic characterization

Plant inoculation and virulence assay were conducted as previously described[49]. In brief, six-week-old cabbage cultivar *Brassica oleraceae* cv. Jingfeng 1 was used as host plants. WT strain of *Xcc* and sterile 10 mM MgCl_2_ were used as positive and negative controls, respectively. All bacterial strains were cultured overnight in NYG medium containing appropriate antibiotics. Cells were collected, washed by 10 mM MgCl_2_, and the concentrations were adjusted to OD_600_ = 0.4 before inoculating into plant leaves using sterile scissors. After inoculation, the plants were kept in a greenhouse at 25°C–30°C and relative humidity >80%. Lesion length was scored 10 days after inoculation, and virulence level was scored semi-quantitatively as follows: 0, no visible effect; 1, limited chlorosis around the cut site; 2, chlorosis extending from the cut site; 3, blackened leaf veins, death, and drying of tissue within the chlorotic area; 4, extensive vein blackening, death, and drying of tissue.

Assay of extracellular polysaccharides production (EPS) was conducted according to previous study[50]. Bacterial strains were cultured at 28°C in NYG medium until OD_600_ = 0.4. If necessary, DSF with appropriate concentration was added and bacteria were cultured for 75–96 hours before EPS production measurement. Quantification of biofilm development was conducted by classic method of crystal violet staining and according to previous study [51]. Bacterial strains were grown at 28°C in NYG medium until OD_600_ = 1.0, and 200 μl culture were inoculated into a 96 well plate (Costar, USA), cultured for 12 hour before quantification. To test the effect of DSF on the formation of biofilm, bacterial strains were cultured and adjusted to OD_600_ = 0.1. Then, those bacteria strains with appropriate concentration of DSF were grown for about 9h at 28°C and 200 μl culture were inoculated into 96 well plate as mentioned above. Estimate of extracellular protease (EXP) were conducted on NYG-milk plate as described previously [52]. If needed, 3.5 μL DSF (30 μM or 10 μM) was added near the bacterial colony.

### Expression and purification of recombinant proteins

C-terminal His_6_-tagged recombinant proteins were expressed by constructing corresponding recombinant pET30a (Novagen) vectors that were electroporated into *E*. *coli* BL21(DE3) strain (S1 Table). Primers used to generate these constructs are listed in S2 Table. His_6_-tagged proteins were expressed and purified using affinity chromatography with Ni-NTA agarose beads (Novagen, USA), according to manufacturer′s instructions. Purified proteins were concentrated using Centricon YM-10 columns (Millipore) and the elute buffer was changed into storage buffer for further use (50 mM Tris-HCl, pH 8.0, 0.5 mM EDTA, 50 mM NaCl and 5% glycerol).

### Preparation and purification of inverted-membrane vesicles

Preparation of inverted membrane vesicles (IMV) containing full-length RpfC was according to the protocol of our previous study with minor modification[53]. Briefly, after sonication, cell debris of *E*. *coli* BL21(DE3) was abandoned by 6,000 g and the membrane containing full-length RpfC in supernatant was collected by ultracentrifugation at 60,000 g at 4°C for 60 min. After ultracentrifugation, the membrane was washed in high-salt buffer (20 mM sodium phosphate, pH 7.0; 2 M KCl; 10% glycerol; 5 mM DTT; 1 mM PMSF) twice. Finally, the membranes were resuspended in 0.5 ml storage buffer (20 mM Tris-HCl, pH 7.5; 10% glycerol) for autokinase assays.

### Reconstruction of membrane-bound histidine kinase by liposome

Liposomes were reconstituted as described by previous study[54]. Briefly, IMV of RpfC was obtained as above-mentioned, and dissolved in suspension buffer (20 mM phosphate, 500 mM NaCl, 20 mM imidazole, pH 7.4) to an approximate concentration of 10 mg ml^-1^ for preparation of liposomes. 900 μl IMV suspension with 100 μl 10% n-Dodecyl-ß-D-maltoside (DDM) were mixed by “end-over-end” mixing for 45 min at 4°C. The supernatant was collected by centrifugation 50,000 g for 30 min and purified by Ni-affinity chromatography. The Ni-NTA beads (Novagen, USA) were pre-equilibrated with 5 volumes of binding buffer (20 mM phosphate, 500 mM NaCl, 20 mM imidazole, pH 7.4, 0.1% DDM). Then the solubilized IMV and pre-equilibrated beads were mixed and incubated at 4°C for 30 min. After the mixing, the supernant was removed and the deposition was washed with binding buffer until the absorbance at OD_280 nm_ returned to base line. Finally, 100 μl of elution buffer (20 mM phosphate, 500 mM NaCl, 250 mM imidazole, pH 7.4, 0.1% DDM) was added to elute the purified RpfC-His_6_. For embedding RpfC by liposome, 10 mg liposomes (Avanti Polar Lipids) were dissolved into 1 ml buffer (50 mM Tris-HCl pH7.5, 10% glycerol, 0.47% Triton-100). Then purified RpfC-His_6_ in elution buffer was added. The mixture was stirred at 4°C for 45 min. The final ratio of phospholipids to protein was about 10:1 (w/w). Bio-Beads (beads: detergent = 10:1, Bio-Rad, USA) were added to remove the detergent and the solution was stirred gently at 4°C overnight. Residual detergent was removed completely by addition of Bio-Beads after further incubation for 2 hours. The Bio-Beads were pipetted off and liposomes containing RpfC-His_6_ were gathered by centrifugation with 200,000 g, 4°C for 30 min. The RpfC liposome was resuspended in final buffer (20 mM Tris-HCl, pH 7.5, 10% glycerol) and stored at 80°C until used.

### *In vitro* phosphorylation assay

*in vitro* phosphorylation assay was conducted as described in our previous study [53]. For autophosphoylation, RpfC liposomes or IMV were incubated with 100 μM ATP containing 10 μCi [γ-^32^P]ATP (PerkinElmer, USA) in autophosphorylation buffer (50 mM Tris-HCl, pH 7.8, 2 mM DTT, 25 mM NaCl, 25 mM KCl, 5 mM MgCl_2_) for indicated time (28°C). If necessary, DSF was added into the mixture 20 min before addition of ATP. The reaction was stopped with 6 × SDS-PAGE loading buffer. The phosphorylated proteins were separated by 12% SDS-PAGE. After SDS-PAGE electrophoresis, gels were separated from back glass plate and placed in a Ziploc bag and exposed to a phosphor screen for 1 hour. The screen was scanned with a PhosphorImage system (Amersham Biosciences, USA) at 50 μM solution. If necessary, signal intensity was measured by Quantity One software (Bio-Rad).

### Purification and on-column cleavage of sensor-GST fusion protein

RpfC sensor fused with a GST tag was obtained and purified according to the GST Gene Fusion System Handbook (Amersham Biosciences) with GST Resin (TRANS). In order to acquire sensor peptide with GST tag cleaved off, on-column cleavage procedure was conducted. In brief, lysate of recombinant *E*.*coli* BL21 (DE3) strain was mixed with pre-equilibrated GST Resin with PBS buffer for ten minutes before loading into column. The column was washed by PBS buffer and resuspened with PreScission buffer (50 mM Tris-HCl, pH 8.5, 150 mM NaCl). Following the injection of 2 units PreScission Protease (GenScript), the column was sealed and placed on a rotator at 4°C. After 10 h of digestion, the flow fractions were collected, which contains preliminary sensor peptide with the GST tag being moved. If necessary, the sensor peptide was purified again by the GST Resin to get rid of uncleaved sensor-GST fusion protein. Eventually, purified sensor peptide was obtained by using size exclusion chromatography with column Superdex 75 10/300 GL (GE Healthcare), stored under -80°C before use.

### Thermal Shift Assay (TSA) using differential scanning fluorimetry

DSF was mixed with purified proteins or liposomes of RpfC to a final concentration of 0 μM, 5 μM or 10 μM respectively in the reaction buffer (50 mM Tris-HCl pH 7.8, 25 mM NaCl, 100 mM KCl). The protein concentration was 0.5–1 μg/μl. The TSA was performed by a Prometheus NT.48 nanoDSF device with a temperature gradient of 20–95°C, 1°C /min. Unfolding transition points were determined according to the changes of intrinsic tryptophan fluorescence at 330 nm, 350 nm. The ratio of fluorescence and the melting temperature (T_m_) were calculated by the NT Melting Control software (NanoTemper Technologies).

### Microscale thermophoresis analysis

Binding reactions of RpfC to DSF was measured by microscale thermophoresis in a Monolith NT.Label Free (Nano Temper Technologies GMBH, Germany) instrument which detects changes in size, charge and conformation induced by binding. RpfC liposomes were collected with centrifugation of 200,000 g for 40 min and resuspended in MST buffer (50 mM Tris-HCl pH 7.8, 150 mM NaCl, 10 mM MgCl_2_, 0.05% Tween-20) to an approximate concentration of 0.1 μM. A range of concentration of DSF (range from 0.06 μM to 2 mM) in assay buffer (50 mM Tris-HCl pH 7.8, 150 mM NaCl, 10 mM MgCl_2_, 0.05% Tween-20, 5% methanol) was incubated with RpfC liposomes (1:1, v/v) for 10 minutes. The sample was loaded into the NT.Label Free standard capillaries and measured with 20% LED power and 40% MST power. Purified sensor protein was dissolved in reaction buffer (50 mM Tris-HCl pH 8.5, 150 mM NaCl, 0.1% Tween-20) to a final concentration as 8 μM. Dilute DSF from 0.0122 μM to 25 μM in buffer (50 mM Tris-HCl pH 8.5, 150 mM NaCl, 0.25‰ methanol). Different concentrations of DSF and sensor protein (1:1, v/v) were mixed and loaded into NT.Label Free standard capillaries. The label free MST assay was performed with 20% LED power and 40% MST power. KD Fit function of the Nano Temper Analysis Software Version 1.5.41 was used to fit curve and calculate the value of dissociation constant (*K*_d_).

### Limited proteolysis

The limited proteolysis experiments were performed at 0°C with 1.4 μg RpfC liposomes in a reaction buffer containing 50 mM Tris-HCl, pH 8.0, 100 mM NaCl, 2 mM DTT and 1.13 mM AMP-PNP. Trypsin was added to a final concentration of 0.018 μg μl^-1^ to degrade RpfC liposome. Aliquot was removed at indicated time and the reaction was stopped by 5 × SDS loading buffer (250 mM Tris-HCl pH 6.8, 10% (w/v) SDS, 0.5% (w/v) bromophenol blue, 50% (v/v) glycerol, 25 mM PMSF). Samples were separated by 12% SDS-PAGE gel and protein bands were detected by silver staining. The sequence of the different peptide fragments were determined by a nanoLC-MS/MS with Orbitrap Fusion system (Thermo scientific, USA).

### Circular Dichroism (CD) analysis

To determine if DSF has impact on RpfC sensor conformation change, CD analysis was carried out on a Chirascan CD Spectrometer (Applied Photophysics, UK), with 10 mm pathlength and 1 nm bandwidth. Sensor protein with GST tag cleaved off was dissolved in buffer (50 mM Tris-HCl pH 8.5, 150 mM NaCl) to 60 μM. Dilute DSF with Sensor protein to a series of concentration (0 μM, 100 μM and 500 μM). CD wavelength scans were collected between 200 nm-260 nm. The spectra data were analyzed on the http://dichroweb.cryst.bbk.ac.uk website with Contin-LL method [55].

## Supporting information

S1 Fig Replacement of the conserved residues had no effect on DSF-RpfC interactions. (A), RpfC^D512V^; (B), RpfC^H657A^. The upper panels show the results of autokinase assays. Each lane contains protein samples that were co-incubated with 100 μM ATP, including 10 μCi [γ-^32^P]ATP, for indicated times. All reactions were immediately stopped using 6 × SDS loading buffer, and separated by 12% SDS-PAGE prior to autoradiography. Band intensities were estimated by Quantity One software and are listed below the panel. The lower panels show proteins stained with Coomassie brilliant blue, which were used as loading controls. Each experiment was independently repeated three times, and a representative experiment is shown.
(PDF)

S2 Fig Stability of RpfC in various bacterial mutants. (A) Stability of RpfC in strains with mutations in both *rpfF* and various input regions of *rpfC*. All strain names containing GUS show that the strain contains a P*engXcc-GUS* biosensor provided in trans by the pHM2 vector. Full-length RpfC with C-terminal His_6_ tag embedded in inverted membrane vesicles (IMV) (upper panel, lane 2) was used as a positive control. Total proteins (T), membrane proteins (M), and soluble cytoplasmic proteins (S) from various bacterial strains were extracted, fractionated, and separated by 12% SDS-PAGE. Upper and middle panels: RpfC in different cellular fractions. Lower panel: loading control. (B-I) Stability of various RpfC proteins in Δ*rpfF*Δ*rpfC* double mutant background. *rpfC* and its various point-mutated forms were provided in trans by recombinant pHM1 vectors. Total proteins were extracted for western blotting. (J-K) Stability of various RpfC proteins in an Δ*rpfC* mutant background. *rpfC* and its various point-mutated forms were provided in trans by recombinant pHM1 vectors to complement the Δ*rpfC* mutant. Total proteins were extracted for western blotting. Western blotting was used to measure the amount of RpfC. Polyclonal antibodies against RpfC (α-RpfC) and RNA polymerase (α-RNAP) from *X*. *campestris* pv. *campestris* were used in western blotting to measure the amount of total proteins, and to compare sample loading. Each experiment was repeated independently three times. Samples were separated on a 12% SDS-PAGE gel and signals were detected using an EZ-ECL enhanced chemiluminescence kit.
(PDF)

S3 Fig Virulence scale of bacterial strains against host cabbage. (A) Quantification of virulence scores showed in Fig 3A. Strain of Δ*rpfC*-*rpfC* was used as positive control. (B and C) Quantification of virulence showed in Fig 5G. Strain of Δ*rpfC*-*rpfC* was used as positive control. (D) Quantification of virulence showed in Fig 8H. Strains used as control in comparison were showed in the panel. In (A-D) Eight-week old cabbage (*Brassica oleraceae* cv. Jingfeng No. 1) was used as host plants. Average virulence scores were estimated 10 days after inoculation (*n* = 16). Standard deviation was showed as vertical bar. * indicates significant difference compared with control strain, calculated by Student’s t-test (*P* < 0.05). BV: Δ*rpfC* strain containing a blank pHM1 vector (A, B, and C) or Δ*rpfF*Δ*rpfC* strain containing a blank pHM1 vector (D).
(PDF)

S4 Fig Substitution of essential amino acid impact DSF-RpfC interaction. Microscale thermophoresis (MST) was used to quantify the binding affinity between DSF and RpfC derivates. Substitutions in RpfC are: (A) S3A; (B) R15D; (C) D17A; (D) S18A; (E) E19A and (F) Q22A. RpfC liposomes were incubated together with DSF in NT Label-free standard capillary in MST assay. Ttrations of DSF ranged from 0.08 to 2500 μM. The solid curve is the fit of the data points to the standard KD-Fit function. Each binding assay was repeated independently three times, and black bars represent standard deviations. *K*_d_ = dissociation constant.
(PDF)

S5 Fig Replacements of essential residues affects DSF-triggered RpfC conformational change. Limited proteolysis was used to analyze interactions between DSF and RpfC liposomes with corresponding replacement. SDS-PAGE of samples and molecular weight analysis revealed DSF-dependent changes in the trypsinolysis pattern, as indicated by asterisks. Replacements in RpfC are: (A) S3A; (B) D17A; (C) S18A; and (D) E19A. Each lane contains 0.32 μg RpfC liposome and 1.13 mM non-hydrolyzable ATP analog adenosine 5′(β,γ-imido) triphosphate (AMP-PNP) as a mimic for nucleotide binding. Each experiment was independently repeated three times and a representative experiment is shown.
(PDF)

S1 Table Bacterial strains and plasmids used in this study. (PDF)

S2 Table Primers used in this study. (PDF)
