# Supplementary material for: Fatty acid DSF binds and allosterically activates histidine kinase RpfC of phytopathogenic bacterium Xanthomonas campestris pv. campestris to regulate quorum-sensing and virulence
Source: PLoS Pathog. 2017 Apr 3;13(4):e1006304. doi: 10.1371/journal.ppat.1006304 (PMC5391125; doi:10.1371/journal.ppat.1006304)
Supplement: S1 Fig — (A), RpfCD512V; (B), RpfCH657A. The upper panels show the results of autokinase assays. Each lane contains protein samples that were co-incubated with 100 μM ATP, including 10 μCi [γ-32P]ATP, for indicated times. All reactions were immediately stopped using 6 × SDS loading buffer, and separated by 12% SDS-PAGE prior to autoradiography. Band intensities were estimated by Quantity One software and are listed below the panel. The lower panels show proteins stained with Coomassie brilliant blue, which were used as loading controls. Each experiment was independently repeated three times, and a representative experiment is shown. (PDF) [file ppat.1006304.s001.pdf]

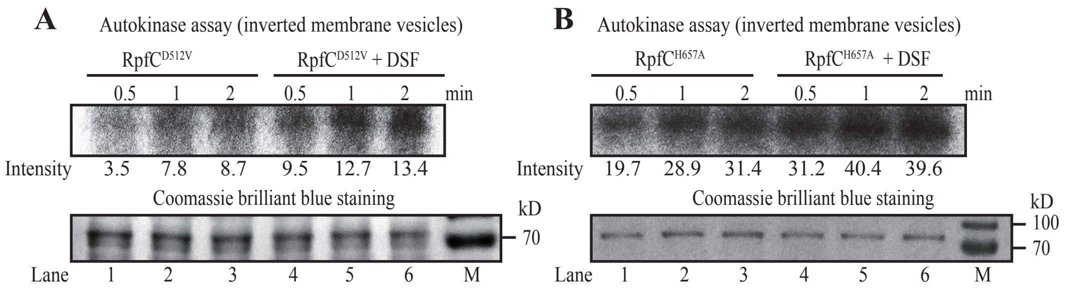

S1 Fig. Replacement of the conserved residues had no effect on DSF-RpfC interactions. (A), RpfC^{D512V}; (B), RpfC^{H657A}. The upper panels show the results of autokinase assays. Each lane contains protein samples that were co-incubated with 100 μM ATP, including 10 μCi [γ-^{32}P]ATP, for indicated times. All reactions were immediately stopped using 6× SDS loading buffer, and separated by 12% SDS-PAGE prior to autoradiography. Band intensities were estimated by Quantity One software and are listed below the panel. The lower panels show proteins stained with Coomassie brilliant blue, which were used as loading controls. Each experiment was independently repeated three times, and a representative experiment is shown.
