# Supplementary material for: Fatty acid DSF binds and allosterically activates histidine kinase RpfC of phytopathogenic bacterium Xanthomonas campestris pv. campestris to regulate quorum-sensing and virulence
Source: PLoS Pathog. 2017 Apr 3;13(4):e1006304. doi: 10.1371/journal.ppat.1006304 (PMC5391125; doi:10.1371/journal.ppat.1006304)
Supplement: S2 Fig — (A) Stability of RpfC in strains with mutations in both rpfF and various input regions of rpfC. All strain names containing GUS show that the strain contains a PengXcc-GUS biosensor provided in trans by the pHM2 vector. Full-length RpfC with C-terminal His6 tag embedded in inverted membrane vesicles (IMV) (upper panel, lane 2) was used as a positive control. Total proteins (T), membrane proteins (M), and soluble cytoplasmic proteins (S) from various bacterial strains were extracted, fractionated, and separated by 12% SDS-PAGE. Upper and middle panels: RpfC in different cellular fractions. Lower panel: loading control. (B-I) Stability of various RpfC proteins in ΔrpfFΔrpfC double mutant background. rpfC and its various point-mutated forms were provided in trans by recombinant pHM1 vectors. Total proteins were extracted for western blotting. (J-K) Stability of various RpfC proteins in an ΔrpfC mutant background. rpfC and its various point-mutated forms were provided in trans by recombinant pHM1 vectors to complement the ΔrpfC mutant. Total proteins were extracted for western blotting. Western blotting was used to measure the amount of RpfC. Polyclonal antibodies against RpfC (α-RpfC) and RNA polymerase (α-RNAP) from X. campestris pv. campestris were used in western blotting to measure the amount of total proteins, and to compare sample loading. Each experiment was repeated independently three times. Samples were separated on a 12% SDS-PAGE gel and signals were detected using an EZ-ECL enhanced chemiluminescence kit. (PDF) [file ppat.1006304.s002.pdf]

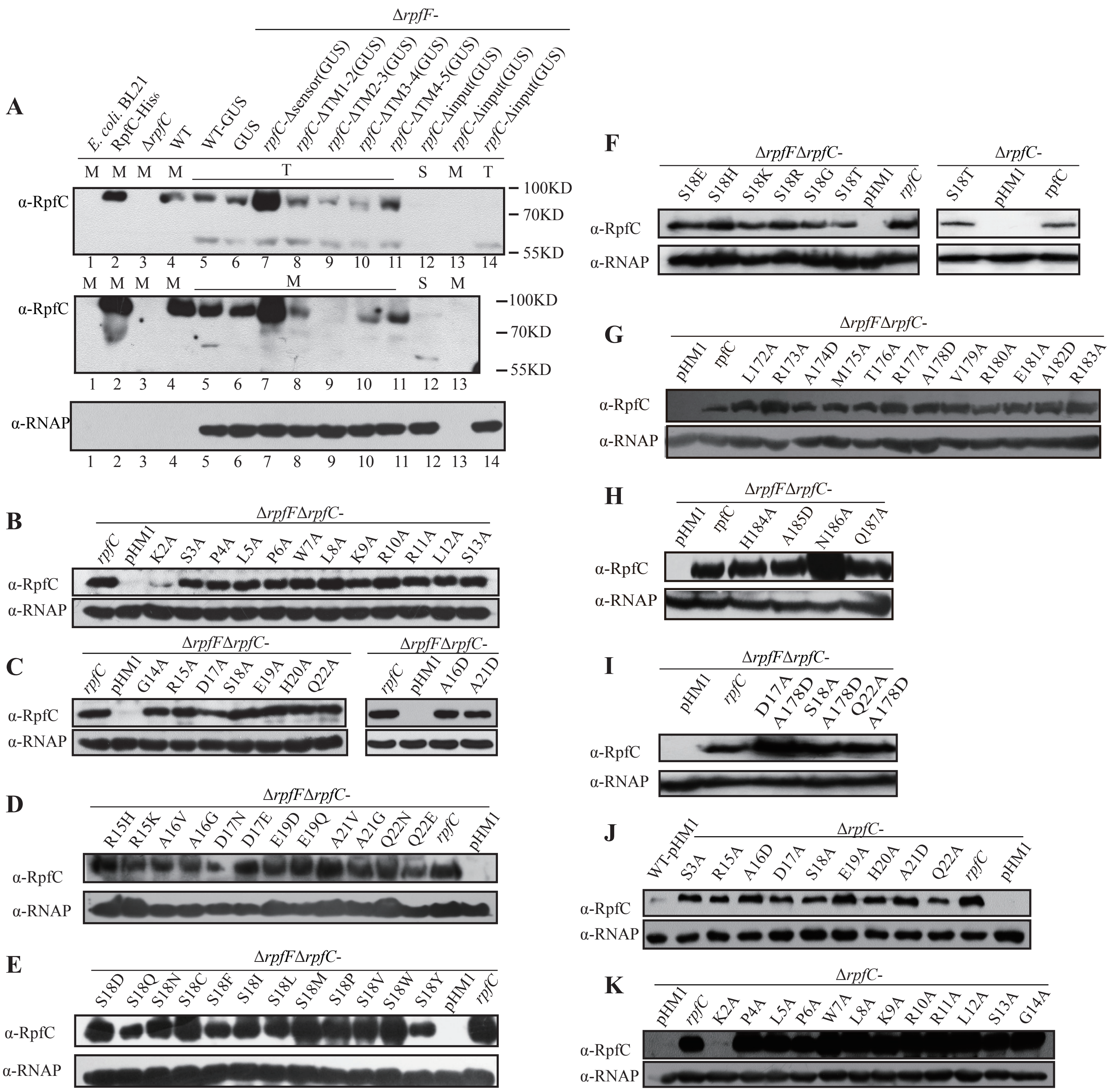

S2 Fig. Stability of RpfC in various bacterial mutants. (A) Stability of RpfC in strains with mutations in both *rpfF* and various input regions of *rpfC*. All strain names containing GUS show that the strain contains a P*engXcc-GUS* biosensor provided *in trans* by the pHM2 vector. Full-length RpfC with C-terminal His_6 tag embedded in inverted membrane vesicles (IMV) (upper panel, lane 2) was used as a positive control. Total proteins (T), membrane proteins (M), and soluble cytoplasmic proteins (S) from various bacterial strains were extracted, fractionated, and separated by 12% SDS-PAGE. Upper and middle panels: RpfC in different cellular fractions. Lower panel: loading control. (B-I) Stability of various RpfC proteins in Δ*rpfF*Δ*rpfC* double mutant mutant background. *rpfC* and its various point-mutated forms were provided *in trans* by recombinant pHM1 vectors. Total proteins were extracted for western blotting. (J-K) Stability of various RpfC proteins in an Δ*rpfC* mutant background. *rpfC* and its various point-mutated forms were provided *in trans* by recombinant pHM1 vectors to complement the Δ*rpfC* mutant. Total proteins were extracted for western blotting. Western blotting was used to measure the amount of RpfC. Polyclonal antibodies against RpfC (α-RpfC) and RNA polymerase (α-RNAP) from *X. campestris* pv. *campestris* were used in western blotting to measure the amount of total proteins, and to compare sample loading. Each experiment was repeated independently three times. Samples were separated on a 12% SDS-PAGE gel and signals were detected using an EZ-ECL enhanced chemiluminescence kit.
