# Supplementary material for: Fatty acid DSF binds and allosterically activates histidine kinase RpfC of phytopathogenic bacterium Xanthomonas campestris pv. campestris to regulate quorum-sensing and virulence
Source: PLoS Pathog. 2017 Apr 3;13(4):e1006304. doi: 10.1371/journal.ppat.1006304 (PMC5391125; doi:10.1371/journal.ppat.1006304)
Supplement: S3 Fig — (A) Quantification of virulence scores showed in Fig 3A. Strain of ΔrpfC-rpfC was used as positive control. (B and C) Quantification of virulence showed in Fig 5G. Strain of ΔrpfC-rpfC was used as positive control. (D) Quantification of virulence showed in Fig 8H. Strains used as control in comparison were showed in the panel. In (A-D) Eight-week old cabbage (Brassica oleraceae cv. Jingfeng No. 1) was used as host plants. Average virulence scores were estimated 10 days after inoculation (n = 16). Standard deviation was showed as vertical bar. * indicates significant difference compared with control strain, calculated by Student’s t-test (P < 0.05). BV: ΔrpfC strain containing a blank pHM1 vector (A, B, and C) or ΔrpfFΔrpfC strain containing a blank pHM1 vector (D). (PDF) [file ppat.1006304.s003.pdf]

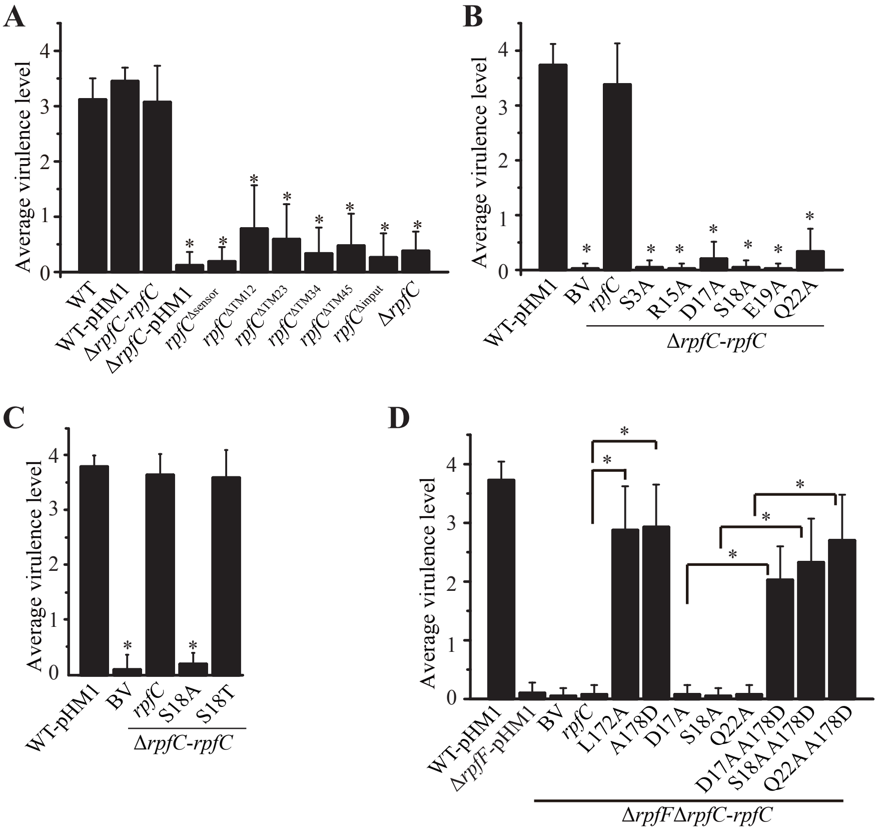

S3 Fig. Virulence scale of bacterial strains against host cabbage. (A) Quantification of virulence scores showed in Fig. 3A. Strain of Δ*rpfC-rpfC* was used as positive control. (B and C) Quantification of virulence showed in Fig. 5G. Strain of Δ *rpfC-rpfC* was used as positive control. (D) Quantification of virulence showed in Fig. 8H. Strains used as control in comparison were showed in the panel. In (A-D) Eight-week old cabbage (*Brassica oleraceae* cv. Jingfeng No. 1) was used as host plants. Average virulence scores were estimated 10 days after inoculation ($n = 16$). Standard deviation was showed as vertical bar. * indicates significant difference compared with control strain, calculated by Student's *t*-test ($P < 0.05$).
