# Supplementary material for: Fatty acid DSF binds and allosterically activates histidine kinase RpfC of phytopathogenic bacterium Xanthomonas campestris pv. campestris to regulate quorum-sensing and virulence
Source: PLoS Pathog. 2017 Apr 3;13(4):e1006304. doi: 10.1371/journal.ppat.1006304 (PMC5391125; doi:10.1371/journal.ppat.1006304)
Supplement: S4 Fig — Microscale thermophoresis (MST) was used to quantify the binding affinity between DSF and RpfC derivates. Substitutions in RpfC are: (A) S3A; (B) R15D; (C) D17A; (D) S18A; (E) E19A and (F) Q22A. RpfC liposomes were incubated together with DSF in NT Label-free standard capillary in MST assay. Ttrations of DSF ranged from 0.08 to 2500 μM. The solid curve is the fit of the data points to the standard KD-Fit function. Each binding assay was repeated independently three times, and black bars represent standard deviations. Kd = dissociation constant. (PDF) [file ppat.1006304.s004.pdf]

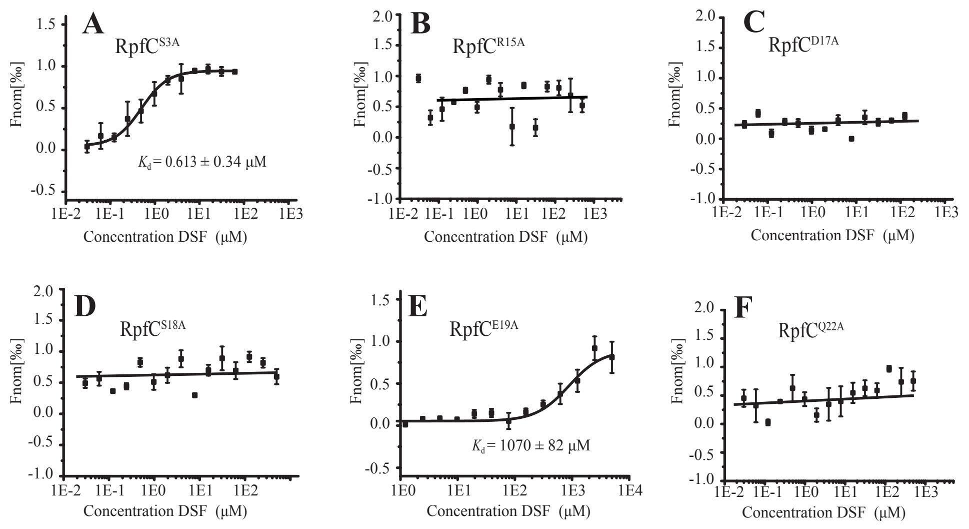

S4 Fig. Substitution of essential amino acid impact DSF-RpfC interaction. Microscale thermophoresis (MST) was used to quantify the binding affinity between DSF and liposome of RpfC derivates. Substitutions in RpfC are: (A) S3A; (B) R15D; (C) D17A; (D) S18A; (E) E19A and (F) Q22A. RpfC liposomes were incubated together with DSF in NT Label-free standard capillary in MST assay. Ttrations of DSF ranged from 0.08 to 2500 μM. The solid curve is the fit of the data points to the standard KD-Fit function. Each binding assay was repeated independently three times, and black bars represent standard deviations. K_d = dissociation constant.
