# Supplementary material for: Fatty acid DSF binds and allosterically activates histidine kinase RpfC of phytopathogenic bacterium Xanthomonas campestris pv. campestris to regulate quorum-sensing and virulence
Source: PLoS Pathog. 2017 Apr 3;13(4):e1006304. doi: 10.1371/journal.ppat.1006304 (PMC5391125; doi:10.1371/journal.ppat.1006304)
Supplement: S5 Fig — Limited proteolysis was used to analyze interactions between DSF and RpfC liposomes with corresponding replacement. SDS-PAGE of samples and molecular weight analysis revealed DSF-dependent changes in the trypsinolysis pattern, as indicated by asterisks. Replacements in RpfC are: (A) S3A; (B) D17A; (C) S18A; and (D) E19A. Each lane contains 0.32 μg RpfC liposome and 1.13 mM non-hydrolyzable ATP analog adenosine 5′(β,γ-imido) triphosphate (AMP-PNP) as a mimic for nucleotide binding. Each experiment was independently repeated three times and a representative experiment is shown. (PDF) [file ppat.1006304.s005.pdf]

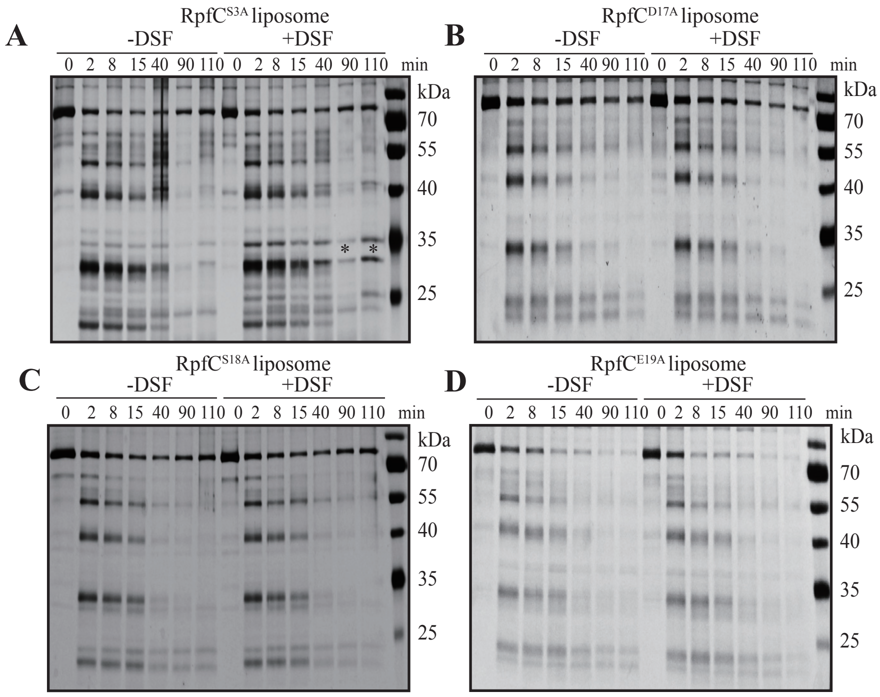

S5 Fig. Replacements of essential residues affects DSF-triggered RpfC conformational change. Limited proteolysis was used to analyze interactions between DSF and RpfC liposomes with corresponding replacement. SDS-PAGE of samples and molecular weight analysis revealed DSF-dependent changes in the trypsinolysis pattern, as indicated by asterisks. Replace ments in RpfC are: (A) S3A; (B) D17A; (C) S18A; and (D) E19A. Each lane contains 0.32 µg RpfC liposome and 1.13 mM non-hydrolyzable ATP analog adenosine 5′(β,γ-imido) triphosphate (AMP-PNP) as a mimic for nucleotide binding. Each experiment was independently repeated three times and a representative experiment is shown.
