# Supplementary material for: Fatty acid DSF binds and allosterically activates histidine kinase RpfC of phytopathogenic bacterium Xanthomonas campestris pv. campestris to regulate quorum-sensing and virulence
Source: PLoS Pathog. 2017 Apr 3;13(4):e1006304. doi: 10.1371/journal.ppat.1006304 (PMC5391125; doi:10.1371/journal.ppat.1006304)
Supplement: S1 Table — (PDF) [file ppat.1006304.s006.pdf]

S1 Table. Bacterial strains and plasmids used in this study

Strain or plasmid	Genotype or description*	Source
**Bacterial strains**		
Escherichia coli strains		
E. coli DH5α	*fhuA2 Δ(argF-lacZ)U169 phoA glnV44 Φ80 Δ(lacZ)M15 gyrA96 recA1 relA1 endA1 thi-1 hsdR17*	Lab collection
E. coli BL21(DE3)	*E. coli B F⁻ dcm ompT hsd*(r_B^- m_B^-) *gal λ(DE3)*	Lab collection
X. campestrips* pv. *campestris strains		
Xcc 8004	Wild type strain (WT), Rif^r	Lab collection
M0033	Δ*rpfC, XC2333* (*rpfC*) in-frame deletion mutant, Rif^r	This study
M0034	Δ*rpfF, XC2332* (*rpfF*) in-frame deletion mutant, Rif^r	This study
M0035	Δ*rpfF*Δ*rpfC, rpfF* and *rpfC* double mutant, Rif^r	This study
M0036	Δ*rpfC-rpfC,* M0033 (*rpfC mutant*) containing a recombinant vector pHM1:: *rpfC,* Rif^r, Sp^r	This study
M0037	*rpfC*$^{\Delta sensor}$, *rpfC* in-frame deletion mutant with 2-22 aa. coding sequences deleted, Rif^r	This study
M0038	*rpfC*$^{\Delta TM1\text{-}2}$, *rpfC* in-frame deletion mutant with 23-72 aa. coding sequences deleted, Rif^r	This study
M0039	*rpfC*$^{\Delta TM2\text{-}3}$, *rpfC* in-frame deletion mutant with 50-115 aa. coding sequences deleted, Rif^r	This study
M0040	*rpfC*$^{\Delta TM3\text{-}4}$, *rpfC* in-frame deletion mutant with 93-147 aa. coding sequences deleted, Rif^r	This study
M0041	*rpfC*$^{\Delta TM4\text{-}5}$, *rpfC* in-frame deletion mutant with 125-171 aa. coding sequences deleted, Rif^r	This study
M0042	*rpfC*$^{\Delta input}$, *rpfC* in-frame deletion mutant with 2-171 aa. coding sequences deleted, Rif^r	This study
M0043	Δ*rpfF*Δ*rpfC-rpfC,* M0035 (*rpfF and rpfC* double in-frame deletion mutant) containing a recombinant vector pHM1:: *rpfC,* Rif^r, Sp^r	This study
M0044	Δ*rpfF-rpfC*$^{\Delta sensor}$*, rpfF* and *rpfC*$^{\Delta sensor}$ double in-frame deletion mutant, Rif^r	This study
M0045	Δ*rpfF-rpfC*$^{\Delta TM1\text{-}2}$*, rpfF* and *rpfC*$^{\Delta TM1\text{-}2}$ double in-frame deletion mutant, Rif^r	This study
M0045	Δ*rpfF-rpfC*$^{\Delta TM2\text{-}3}$*, rpfF* and *rpfC*$^{\Delta TM2\text{-}3}$ double in-frame deletion mutant, Rif^r	This study
M0046	Δ*rpfF-rpfC*$^{\Delta TM34}$*, rpfF* and *rpfC*$^{\Delta TM34}$ double in-frame deletion mutant, Rif^r	This study
M0047	Δ*rpfF-rpfC*$^{\Delta TM4\text{-}5}$*, rpfF* and *rpfC*$^{\Delta TM4\text{-}5}$ double in-frame deletion mutant, Rif^r	This study
M0048	Δ*rpfF-rpfC*$_{\Delta input}$*, rpfF* and *rpfC*$_{\Delta input}$ double in-frame deletion mutant, Rif^r	This study
M0049	Δ*rpfF*(*GUS*), Δ*rpfF* derivate containing a recombinant vector pHM2:: P*engXcc-GUS,* Rif^r, Sp^r	This study
M0050	*rpfC*$^{\Delta sensor}$(*GUS*), *rpfC*$^{\Delta sensor}$ derivate containing a	This study

	recombinant vector pHM2:: P*engXcc-GUS,* Rif^r, Sp^r	
M0051	*rpfC*$^{\Delta TM1-2}$(*GUS*), *rpfC*$^{\Delta TM1-2}$ derivate containing a recombinant vector pHM2:: P*engXcc-GUS,* Rif^r, Sp^r	This study
M0052	*rpfC*$^{\Delta TM2-3}$(*GUS*), *rpfC*$^{\Delta TM2-3}$ derivate containing a recombinant vector pHM2:: P*engXcc-GUS,* Rif^r, Sp^r	This study
M0053	*rpfC*$^{\Delta TM3-4}$(*GUS*), *rpfC*$^{\Delta TM3-4}$ derivate containing a recombinant vector pHM2:: P*engXcc-GUS,* Rif^r, Sp^r	This study
M0054	*rpfC*$^{\Delta TM4-5}$(*GUS*), *rpfC*$^{\Delta TM4-5}$ derivate containing a recombinant vector pHM2:: P*engXcc-GUS,* Rif^r, Sp^r	This study
M0055	*rpfC*$^{\Delta input}$(*GUS*), *rpfC*$^{\Delta input}$ derivate containing a recombinant vector pHM2:: P*engXcc-GUS,* Rif^r, Sp^r	This study
M0056	Δ*rpfF*Δ*rpfC*(*GUS*), Δ*rpfF*Δ*rpfC* derivate containing a recombinant vector pHM2:: P*engXcc-GUS,* Rif^r, Sp^r	This study
M0057	Δ*rpfF- rpfC*$^{\Delta sensor}$(*GUS*), Δ*rpfF- rpfC*$_{\Delta sensor}$ derivate containing a recombinant vector pHM2:: P*engXcc-GUS,* Rif^r, Sp^r	This study
M0058	Δ*rpfF- rpfC*$^{\Delta TM1-2}$(*GUS*), *ΔrpfF- rpfC*$^{\Delta TM1-2}$ derivate containing a recombinant vector pHM2:: P*engXcc-GUS,* Rif^r, Sp^r	This study
M0059	Δ*rpfF- rpfC*$^{\Delta TM3-4}$(*GUS*), Δ*rpfF- rpfC*$^{\Delta TM3-4}$ derivate containing a recombinant vector pHM2:: P*engXcc-GUS,* Rif^r, Sp^r	This study
M0060	Δ*rpfF- rpfC*$^{\Delta TM4-5}$(*GUS*), Δ*rpfF- rpfC*$^{\Delta TM4-5}$ derivate containing a recombinant vector pHM2:: P*engXcc-GUS,* Rif^r, Sp^r	This study
M0061	Δ*rpfF-GUS,* transcriptional fusion. a *gusE* gene and its own SD sequence was fused into the DNA region between P*engXcc* promoter and *engXcc* in the background of Δ*rpfF*	This study
M0062	Δ*rpfF*Δ*rpfC-GUS,* transcriptional fusion. a *gusE* gene and its own SD sequence was fused into the DNA region between Peng*Xcc* promoter and *engXcc* in the background of Δ*rpfF*-Δ*rpfC*	This study
M0063	Δ*rpfF*Δ*rpfC-rpfC*^{K2A}*-GUS,* M0062 containing a recombinant vector pHM1:: *rpfC*^{K2A}, Rif^r, Sp^r	This study
M0064	Δ*rpfF*Δ*rpfC-rpfC*^{S3A}*-GUS,* M0062 containing a recombinant vector pHM1:: *rpfC*^{S3A}, Rif^r, Sp^r	This study
M0065	Δ*rpfF*Δ*rpfC-rpfC*^{P4A}*-GUS,* M0062 containing a recombinant vector pHM1:: *rpfC*^{P4A}, Rif^r, Sp^r	This study
M0066	Δ*rpfF*Δ*rpfC-rpfC*^{L5A}*-GUS,* M0062 containing a recombinant vector pHM1:: *rpfC*^{L5A}, Rif^r, Sp^r	This study
M0067	Δ*rpfF*Δ*rpfC-rpfC*^{P6A}*-GUS,* M0062 containing a recombinant vector pHM1:: *rpfC*^{P6A}, Rif^r, Sp^r	This study
M0068	Δ*rpfF*Δ*rpfC-rpfC*^{W7A}*-GUS,* M0062 containing a recombinant vector pHM1:: *rpfC*^{W7A}, Rif^r, Sp^r	This study
M0069	Δ*rpfF*Δ*rpfC-rpfC*^{L8A}*-GUS,* M0062 containing a recombinant	This study

	vector pHM1:: *rpfC*^{L8A}, Rif^{r}, Sp^{r}	
M0070	Δ*rpfF*Δ*rpfC-rpfC*^{K9A}-*GUS,* M0062 containing a recombinant vector pHM1:: *rpfC*^{K9A}, Rif^{r}, Sp^{r}	This study
M0071	Δ*rpfF*Δ*rpfC-rpfC*^{R10A}-*GUS,* M0062 containing a recombinant vector pHM1:: *rpfC*^{R10A}, Rif^{r}, Sp^{r}	This study
M0072	Δ*rpfF*Δ*rpfC-rpfC*^{R11A}-*GUS,* M0062 containing a recombinant vector pHM1:: *rpfC*^{R11A}, Rif^{r}, Sp^{r}	This study
M0073	Δ*rpfF*Δ*rpfC-rpfC*^{L12A}-*GUS,* M0062 containing a recombinant vector pHM1:: *rpfC*^{L12A}, Rif^{r}, Sp^{r}	This study
M0074	Δ*rpfF*Δ*rpfC-rpfC*^{S13A}-*GUS,* M0062 containing a recombinant vector pHM1:: *rpfC*^{S13A}, Rif^{r}, Sp^{r}	This study
M0075	Δ*rpfF*Δ*rpfC-rpfC*^{G14A}-*GUS,* M0062 containing a recombinant vector pHM1:: *rpfC*^{G14A}, Rif^{r}, Sp^{r}	This study
M0076	Δ*rpfF*Δ*rpfC-rpfC*^{R15A}-*GUS,* M0062 containing a recombinant vector pHM1:: *rpfC*^{R15A}, Rif^{r}, Sp^{r}	This study
M0077	Δ*rpfF*Δ*rpfC-rpfC*^{A16D}-*GUS,* M0062 containing a recombinant vector pHM1:: *rpfC*^{A16D}, Rif^{r}, Sp^{r}	This study
M0078	Δ*rpfF*Δ*rpfC-rpfC*^{D17A}-*GUS,* M0062 containing a recombinant vector pHM1:: *rpfC*^{D17A}, Rif^{r}, Sp^{r}	This study
M0079	Δ*rpfF*Δ*rpfC-rpfC*^{S18A}-*GUS,* M0062 containing a recombinant vector pHM1:: *rpfC*^{S18A}, Rif^{r}, Sp^{r}	This study
M0080	Δ*rpfF*Δ*rpfC-rpfC*^{E19A}-*GUS,* M0062 containing a recombinant vector pHM1:: *rpfC*^{E19A}, Rif^{r}, Sp^{r}	This study
M0081	Δ*rpfF*Δ*rpfC-rpfC*^{H20A}-*GUS,* M0062 containing a recombinant vector pHM1:: *rpfC*^{H20A}, Rif^{r}, Sp^{r}	This study
M0082	Δ*rpfF*Δ*rpfC-rpfC*^{A21D}-*GUS,* M0062 containing a recombinant vector pHM1:: *rpfC*^{A21D}, Rif^{r}, Sp^{r}	This study
M0083	Δ*rpfF*Δ*rpfC-rpfC*^{Q22A}-*GUS,* M0062 containing a recombinant vector pHM1:: *rpfC*^{Q22A}, Rif^{r}, Sp^{r}	This study
M0084	Δ*rpfF*Δ*rpfC-rpfC-GUS,* M0062 containing a recombinant vector pHM1:: *rpfC,* Rif^{r}, Sp^{r}	This study
M0085	Δ*rpfC-rpfC*^{K2A}, M0033 containing a recombinant vector pHM1:: *rpfC*^{K2A}, Rif^{r}, Sp^{r}	This study
M0086	Δ*rpfC-rpfC*^{S3A}, M0033 containing a recombinant vector pHM1:: *rpfC*^{S3A}, Rif^{r}, Sp^{r}	This study
M0087	Δ*rpfC-rpfC*^{P4A}, M0033 containing a recombinant vector pHM1:: *rpfC*^{P4A}, Rif^{r}, Sp^{r}	This study
M0088	Δ*rpfC-rpfC*^{L5A}, M0033 containing a recombinant vector pHM1:: *rpfC*^{L5A}, Rif^{r}, Sp^{r}	This study
M0089	Δ*rpfC-rpfC*^{P6A}, M0033 containing a recombinant vector pHM1:: *rpfC*^{P6A}, Rif^{r}, Sp^{r}	This study
M0090	Δ*rpfC-rpfC*^{W7A}, M0033 containing a recombinant vector pHM1:: *rpfC*^{W7A}, Rif^{r}, Sp^{r}	This study
M0091	Δ*rpfC-rpfC*^{L8A}, M0033 containing a recombinant vector pHM1:: *rpfC*^{L8A}, Rif^{r}, Sp^{r}	This study

M0092	Δ*rpfC-rpfC*^{K9A}, M0033 containing a recombinant vector pHM1:: *rpfC*^{K9A}, Rif^{r}, Sp^{r}	This study
M0093	Δ*rpfC-rpfC*^{R10A}, M0033 containing a recombinant vector pHM1:: *rpfC*^{R10A}, Rif^{r}, Sp^{r}	This study
M0094	Δ*rpfC-rpfC*^{R11A}, M0033 containing a recombinant vector pHM1:: *rpfC*^{R11A}, Rif^{r}, Sp^{r}	This study
M0095	Δ*rpfC-rpfC*^{L12A}, M0033 containing a recombinant vector pHM1:: *rpfC*^{L12A}, Rif^{r}, Sp^{r}	This study
M0096	Δ*rpfC-rpfC*^{S13A}, M0033 containing a recombinant vector pHM1:: *rpfC*^{S13A}, Rif^{r}, Sp^{r}	This study
M0097	Δ*rpfC-rpfC*^{G14A}, M0033 containing a recombinant vector pHM1:: *rpfC*^{G14A}, Rif^{r}, Sp^{r}	This study
M0098	Δ*rpfC-rpfC*^{R15A}, M0033 containing a recombinant vector pHM1:: *rpfC*^{R15A}, Rif^{r}, Sp^{r}	This study
M0099	Δ*rpfC-rpfC*^{A16D}, M0033 containing a recombinant vector pHM1:: *rpfC*^{A16D}, Rif^{r}, Sp^{r}	This study
M0100	Δ*rpfC-rpfC*^{D17A}, M0033 containing a recombinant vector pHM1:: *rpfC*^{D17A}, Rif^{r}, Sp^{r}	This study
M0101	Δ*rpfC-rpfC*^{S18A}, M0033 containing a recombinant vector pHM1:: *rpfC*^{S18A}, Rif^{r}, Sp^{r}	This study
M0102	Δ*rpfC-rpfC*^{E19A}, M0033 containing a recombinant vector pHM1:: *rpfC*^{E19A}, Rif^{r}, Sp^{r}	This study
M0103	Δ*rpfC-rpfC*^{H20A}, M0033 containing a recombinant vector pHM1:: *rpfC*^{H20A}, Rif^{r}, Sp^{r}	This study
M0104	Δ*rpfC-rpfC*^{A21D}, M0033 containing a recombinant vector pHM1:: *rpfC*^{A21D}, Rif^{r}, Sp^{r}	This study
M0105	Δ*rpfC-rpfC*^{Q22A}, M0033 containing a recombinant vector pHM1:: *rpfC*^{Q22A}, Rif^{r}, Sp^{r}	This study
M0106	Δ*rpfF*Δ*rpfC-rpfC*^{R15K}-*GUS,* M0062 containing a recombinant vector pHM1:: *rpfC*^{R15K}, Rif^{r}, Sp^{r}	This study
M0107	Δ*rpfF*Δ*rpfC-rpfC*^{R15H}-*GUS,* M0062 containing a recombinant vector pHM1:: *rpfC*^{R15H}, Rif^{r}, Sp^{r}	This study
M0108	Δ*rpfF*Δ*rpfC-rpfC*^{A16V}-*GUS,* M0062 containing a recombinant vector pHM1:: *rpfC*^{A16V}, Rif^{r}, Sp^{r}	This study
M0109	Δ*rpfF*Δ*rpfC-rpfC*^{A16G}-*GUS,* M0062 containing a recombinant vector pHM1:: *rpfC*^{A16G}, Rif^{r}, Sp^{r}	This study
M0110	Δ*rpfF*Δ*rpfC-rpfC*^{D17E}-*GUS,* M0062 containing a recombinant vector pHM1:: *rpfC*^{D17E}, Rif^{r}, Sp^{r}	This study
M0111	Δ*rpfF*Δ*rpfC-rpfC*^{D17N}-*GUS,* M0062 containing a recombinant vector pHM1:: *rpfC*^{D17N}, Rif^{r}, Sp^{r}	This study
M0112	Δ*rpfF*Δ*rpfC-rpfC*E19D-*GUS,* M0062 containing a recombinant vector pHM1:: *rpfC*^{E19D}, Rif^{r}, Sp^{r}	This study
M0113	Δ*rpfF*Δ*rpfC-rpfC*^{E19Q}-*GUS,* M0062 containing a recombinant vector pHM1:: *rpfC*^{E19Q}, Rif^{r}, Sp^{r}	This study
M0114	Δ*rpfF*Δ*rpfC-rpfC*^{A21V}-*GUS,* M0062 containing a	This study

	recombinant vector pHM1:: *rpfC*^{A21V}, Rif^{r}, Sp^{r}	
M0115	Δ*rpfF*Δ*rpfC*-*rpfC*^{A21G}-*GUS*, M0062 containing a recombinant vector pHM1:: *rpfC*^{A21G}, Rif^{r}, Sp^{r}	This study
M0116	Δ*rpfF*Δ*rpfC*-*rpfC*^{Q22N}-*GUS*, M0062 containing a recombinant vector pHM1:: *rpfC*^{Q22N}, Rif^{r}, Sp^{r}	This study
M0117	Δ*rpfF*Δ*rpfC*-*rpfC*^{Q22E}-*GUS*, M0062 containing a recombinant vector pHM1:: *rpfC*^{Q22E}, Rif^{r}, Sp^{r}	This study
M0118	Δ*rpfF*Δ*rpfC*-*rpfC*^{S18N}-*GUS*, M0062 containing a recombinant vector pHM1:: *rpfC*^{S18N}, Rif^{r}, Sp^{r}	This study
M0119	Δ*rpfF*Δ*rpfC*-*rpfC*^{S18Q}-*GUS*, M0062 containing a recombinant vector pHM1:: *rpfC*^{S18Q}, Rif^{r}, Sp^{r}	This study
M0120	Δ*rpfF*Δ*rpfC*-*rpfC*^{S18D}-*GUS*, M0062 containing a recombinant vector pHM1:: *rpfC*^{S18D}, Rif^{r}, Sp^{r}	This study
M0121	Δ*rpfF*Δ*rpfC*-*rpfC*^{S18E}-*GUS*, M0062 containing a recombinant vector pHM1:: *rpfC*^{S18E}, Rif^{r}, Sp^{r}	This study
M0122	Δ*rpfF*Δ*rpfC*-*rpfC*^{S18K}-*GUS*, M0062 containing a recombinant vector pHM1:: *rpfC*^{S18K}, Rif^{r}, Sp^{r}	This study
M0123	Δ*rpfF*Δ*rpfC*-*rpfC*^{S18R}-*GUS*, M0062 containing a recombinant vector pHM1:: *rpfC*^{S18R}, Rif^{r}, Sp^{r}	This study
M0124	Δ*rpfF*Δ*rpfC*-*rpfC*^{S18H}-*GUS*, M0062 containing a recombinant vector pHM1:: *rpfC*^{S18H}, Rif^{r}, Sp^{r}	This study
M0125	Δ*rpfF*Δ*rpfC*-*rpfC*^{S18G}-*GUS*, M0062 containing a recombinant vector pHM1:: *rpfC*^{S18G}, Rif^{r}, Sp^{r}	This study
M0126	Δ*rpfF*Δ*rpfC*-*rpfC*^{S18V}-*GUS*, M0062 containing a recombinant vector pHM1:: *rpfC*^{S18V}, Rif^{r}, Sp^{r}	This study
M0127	Δ*rpfF*Δ*rpfC*-*rpfC*^{S18L}-*GUS*, M0062 containing a recombinant vector pHM1:: *rpfC*^{S18L}, Rif^{r}, Sp^{r}	This study
M0128	Δ*rpfF*Δ*rpfC*-*rpfC*^{S18I}-*GUS*, M0062 containing a recombinant vector pHM1:: *rpfC*^{S18I}, Rif^{r}, Sp^{r}	This study
M0129	Δ*rpfF*Δ*rpfC*-*rpfC*^{S18P}-*GUS*, M0062 containing a recombinant vector pHM1:: *rpfC*^{S18P}, Rif^{r}, Sp^{r}	This study
M0130	Δ*rpfF*Δ*rpfC*-*rpfC*^{S18F}-*GUS*, M0062 containing a recombinant vector pHM1:: *rpfC*^{S18F}, Rif^{r}, Sp^{r}	This study
M0131	Δ*rpfF*Δ*rpfC*-*rpfC*^{S18Y}-*GUS*, M0062 containing a recombinant vector pHM1:: *rpfC*^{S18Y}, Rif^{r}, Sp^{r}	This study
M0132	Δ*rpfF*Δ*rpfC*-*rpfC*^{S18W}-*GUS*, M0062 containing a recombinant vector pHM1:: *rpfC*^{S18W}, Rif^{r}, Sp^{r}	This study
M0133	Δ*rpfF*Δ*rpfC*-*rpfC*^{S18C}-*GUS*, M0062 containing a recombinant vector pHM1:: *rpfC*^{S18C}, Rif^{r}, Sp^{r}	This study
M0134	Δ*rpfF*Δ*rpfC*-*rpfC*^{S18M}-*GUS*, M0062 containing a recombinant vector pHM1:: *rpfC*^{S18M}, Rif^{r}, Sp^{r}	This study
M0135	Δ*rpfF*Δ*rpfC*-*rpfC*^{S18T}-*GUS*, M0062 containing a recombinant vector pHM1:: *rpfC*^{S18T}, Rif^{r}, Sp^{r}	This study
M0136	Δ*rpfF*Δ*rpfC*-*rpfC*^{L172A}-*GUS*, M0062 containing a recombinant vector pHM1:: *rpfC*^{L172A}, Rif^{r}, Sp^{r}	This study

M0137	Δ*rpfF*Δ*rpfC-rpfC*^{R173A}-*GUS,* M0062 containing a recombinant vector pHM1:: *rpfC*^{R173A}, Rif^{r}, Sp^{r}	This study
M0138	Δ*rpfF*Δ*rpfC-rpfC*^{A174D}-*GUS,* M0062 containing a recombinant vector pHM1:: *rpfC*^{A174D}, Rif^{r}, Sp^{r}	This study
M0139	Δ*rpfF*Δ*rpfC-rpfC*^{M175A}-*GUS,* M0062 containing a recombinant vector pHM1:: *rpfC*^{M175A}, Rif^{r}, Sp^{r}	This study
M0140	Δ*rpfF*Δ*rpfC-rpfC*^{T176A}-*GUS,* M0062 containing a recombinant vector pHM1:: *rpfC*^{T176A}, Rif^{r}, Sp^{r}	This study
M0141	Δ*rpfF*Δ*rpfC-rpfC*^{R177A}-*GUS,* M0062 containing a recombinant vector pHM1:: *rpfC*^{R177A}, Rif^{r}, Sp^{r}	This study
M0142	Δ*rpfF*Δ*rpfC-rpfC*^{A178D}-*GUS,* M0062 containing a recombinant vector pHM1:: *rpfC*^{A178D}, Rif^{r}, Sp^{r}	This study
M0143	Δ*rpfF*Δ*rpfC-rpfC*^{V179A}-*GUS,* M0062 containing a recombinant vector pHM1:: *rpfC*^{V179A}, Rif^{r}, Sp^{r}	This study
M0144	Δ*rpfF*Δ*rpfC-rpfC*^{R180A}-*GUS,* M0062 containing a recombinant vector pHM1:: *rpfC*^{R180A}, Rif^{r}, Sp^{r}	This study
M0145	Δ*rpfF*Δ*rpfC-rpfC*^{E181A}-*GUS,* M0062 containing a recombinant vector pHM1:: *rpfC*^{E181A}, Rif^{r}, Sp^{r}	This study
M0146	Δ*rpfF*Δ*rpfC-rpfC*^{A182D}-*GUS,* M0062 containing a recombinant vector pHM1:: *rpfC*^{A182D}, Rif^{r}, Sp^{r}	This study
M0147	Δ*rpfF*Δ*rpfC-rpfC*^{R183A}-*GUS,* M0062 containing a recombinant vector pHM1:: *rpfC*^{R183A}, Rif^{r}, Sp^{r}	This study
M0148	Δ*rpfF*Δ*rpfC-rpfC*^{H184A}-*GUS,* M0062 containing a recombinant vector pHM1:: *rpfC*^{H184A}, Rif^{r}, Sp^{r}	This study
M0149	Δ*rpfF*Δ*rpfC-rpfC*^{A185D}-*GUS,* M0062 containing a recombinant vector pHM1:: *rpfC*^{A185D}, Rif^{r}, Sp^{r}	This study
M0150	Δ*rpfF*Δ*rpfC-rpfC*^{N186A}-*GUS,* M0062 containing a recombinant vector pHM1:: *rpfC*^{N186A}, Rif^{r}, Sp^{r}	This study
M0151	Δ*rpfF*Δ*rpfC-rpfC*^{Q187A}-*GUS,* M0062 containing a recombinant vector pHM1:: *rpfC*^{Q187A}, Rif^{r}, Sp^{r}	This study
M0152	Δ*rpfF*Δ*rpfC-rpfC*$^{D17A-A178D}$-*GUS,* M0062 containing a recombinant vector pHM1:: *rpfC*$^{D17A-A178D}$, Rif^{r}, Sp^{r}	This study
M0153	Δ*rpfF*Δ*rpfC-rpfC*$^{S18A-A178D}$-*GUS,* M0062 containing a recombinant vector pHM1:: *rpfC*$^{S18A-A178D}$, Rif^{r}, Sp^{r}	This study
M0154	Δ*rpfF*Δ*rpfC-rpfC*$^{Q22A-A178D}$-*GUS,* M0062 containing a recombinant vector pHM1:: *rpfC*$^{Q22A-A178D}$, Rif^{r}, Sp^{r}	This study
M0155	Δ*rpfC-rpfC*^{S18T}, M0033 containing a recombinant vector pHM1:: *rpfC*^{S18T}, Rif^{r}, Sp^{r}	This study
**Plasmids**		
pK18mob	Suicide vector used in single-crossover recombination, Kan^{r}	(1)
pK18mobsacB	Suicide vector used in double-crossover recombination, Kan^{r}	(1)
pHM1	Complementary vector with P*lac* promoter before MCS, Sp^{r}	(2)
pHM2	Complementary vector with no promoter before MCS, Sp^{r}	Lab collection

pET30a	Protein expression vector, Kan^r	Novagen
pGEX-6P-1	Protein expression vector, Amp^r	Lab collection
pET30a-RpfC	Protein expression vector, pET30a::RpfC, Kan^r, expressing full-length RpfC	This study
pET30a-$RpfC_{\Delta input}$	Protein expression vector，pET30a::$RpfC^{\Delta input}$, Kan^r, expressing RpfC without input domain	This study
pET30a-RpfC-H657A	Protein expression vector，pET30a::$RpfC^{H657A}$, Kan^r, expressing the H198A point mutated protein of RpfC	This study
pET30a-RpfC-D512V	Protein expression vector，pET30a::$RpfC^{D512V}$, Kan^r, expressing the D512V point mutated protein of RpfC	This study
pET30a-RpfC-S3A	Protein expression vector，pET30a::$RpfC^{S3A}$, Kan^r, expressing the S3A point mutated protein of RpfC	This study
pET30a-RpfC-R15A	Protein expression vector，pET30a::$RpfC^{R15A}$, Kan^r, expressing the R15A point mutated protein of RpfC	This study
pET30a-RpfC-A16D	Protein expression vector，pET30a::$RpfC^{A16D}$, Kan^r, expressing the A16D point mutated protein of RpfC	This study
pET30a-RpfC-D17A	Protein expression vector，pET30a::$RpfC^{D17A}$, Kan^r, expressing the D17A point mutated protein of RpfC	This study
pET30a-RpfC-S18A	Protein expression vector，pET30a::$RpfC^{S18A}$, Kan^r, expressing the S18A point mutated protein of RpfC	This study
pET30a-RpfC-E19A	Protein expression vector，pET30a::$RpfC^{E19A}$, Kan^r, expressing the E19A point mutated protein of RpfC	This study
pET30a-RpfC-H20A	Protein expression vector，pET30a::$RpfC^{H20A}$, Kan^r, expressing the H20A point mutated protein of RpfC	This study
pET30a-RpfC-A21D	Protein expression vector，pET30a::$RpfC^{A21D}$, Kan^r, expressing the A21D point mutated protein of RpfC	This study
pET30a-RpfC-Q22A	Protein expression vector，pET30a::$RpfC^{Q22A}$, Kan^r, expressing the Q22A point mutated protein of RpfC	This study
pET30a-RpfC-S18T	Protein expression vector，pET30a::$RpfC^{S18T}$, Kan^r, expressing the S18T point mutated protein of RpfC	This study
Sensor-GST	Protein expression vector，pGEX-6P-1::sensor, Amp^r, expressing sensor region of RpfC, Amp^r	This study
pET30a-$RpfC_{\Delta}$sensor	Protein expression vector，pET30a::$RpfC_{\Delta}$sensor, Kan^r ,expressing full length of RpfC with 22 aa of N terminal deletion	This study
pET30a-RpfG	Protein expression vector, pET30a::RpfG, Kan^r, expressing full-length RpfG	This study
pET30a-RpfG-D80V	Protein expression vector，pET30a::$RpfG^{D80V}$, Kan^r, expressing the D80V point mutated protein of RpfG.	This study

* SD sequence: The Shine-Dalgarno (SD) sequence. MCS site: multiple cloning site. Kan^r: resistant to kanamycin; Raf^r: resistant to rifamycin; Sp^r: resistant to spectinomycin.

References

1. Schafer A, *et al.* (1994) Small mobilizable multi-purpose cloning vectors derived from the *Escherichia coli* plasmids pK18 and pK19: selection of defined deletions in

the chromosome of *Corynebacterium glutamicum*. *Gene* 145(1):69-73.

2. Innes RW, Hirose MA, Kuempel PL (1988) Induction of nitrogen-fixing nodules on clover requires only 32 kilobase pairs of DNA from the *Rhizobium trifolii* symbiosis plasmid. *J Bacteriol* 170(9):3793-3802.
