# Supplementary material for: Fatty acid DSF binds and allosterically activates histidine kinase RpfC of phytopathogenic bacterium Xanthomonas campestris pv. campestris to regulate quorum-sensing and virulence
Source: PLoS Pathog. 2017 Apr 3;13(4):e1006304. doi: 10.1371/journal.ppat.1006304 (PMC5391125; doi:10.1371/journal.ppat.1006304)
Supplement: S2 Table — (PDF) [file ppat.1006304.s007.pdf]

S2 Table. Primers used in this study

Primer name	Sequence (forward/reverse)	Length/description
**ΔrpfF**	A: AAGCTTCCCAACGAGATCGAAGAC<br>B: GGATCCGGTGGTTCTCCGTGAGAC<br>C: GGATCCCAGTCGCGTCGCTCAG<br>D: GGCGAGGAATTCGAACGCCAGTTC	AB 446 bp; CD 363 bp;<br>For Δ*rpfF* construction
**ΔrpfC**	A: GAATTCGACAGCGAGCACGGTCAA<br>B: TCTAGAAGACTTCATAGACGCCTCAGAC<br>C: TCTAGATAGACCGCCGGCGCATCA<br>D: AAGCTTGCACTAAGCTGCCACACGATCA	AB 592 bp; CD 459 bp;<br>For Δ*rpfC* construction
**CrpfC**	A:GCAAGCTTATGAAGTCTCCATTGCCATG<br>B: GCGTCGACCTATTCGTTGCTACGGGGGG	2181 bp; for Δ*rpfC-rpfC*, Δ*rpfF*Δ*rpfC-rpfC* construction
**ΔrpfF-ΔrpfC**	A：GAATTCGACAGCGAGCACGGTCAA<br>B: TCTAGAAGACTTCATAGACGCCTCAGAC<br>C:GCTCTAGATGAGGTGGTTCTCCGTGAGAC<br>D: AAGCTTCCCAACGAGATCGAAGAC	AB 592 bp; CD 446 bp;<br>For Δ*rpfF*-Δ*rpfC* construction
**P*engXcc*-*GUS*-pHM2**	A:GCGTCGACTTCGCGCGCAGGCGAG<br>B: GCGGATCCAGAGAACCGGGCTCGAC	254 bp; for pHM2:: *engXcc-GUS* construction
**ΔrpfFΔrpfC GUS**	A:GCGTCGACTTCGCGCGCAGGCGAG<br>B: GCGGATCCAGAGAACCGGGCTCGAC<br>C:GCGGTACCAGGGAGATCACCATGTC<br>D: GCAAGCTTGCATCTGCACGATCATGTCC	AB 254 bp; CD 235 bp; For Δ*rpfF*Δ*rpfC*-GUS construction
**Δsensor**	A: GCGAATTC GTGCTGATCTTTTGCCGG<br>B: GCCATATG CATAGACGCCTCAGACGCGC<br>C: GCCATATG AATCTGATCCGCATCATC<br>D: GCAAGCTT CAATAAGGAGAAATCAGG	AB 439 bp; CD 374 bp; For *rpfC*$^{\Delta sensor}$, Δ*rpfF-rpfC*$^{\Delta sensor}$ construction
**ΔTM12**	A: GCGAATTC GTGCTGATCTTTTGCCGG<br>B: GCCATATG CTGCGCGTGTTCCGAATC<br>C: GCCATATG CGACCGCAGGTGTCGCATAC<br>D: GCAAGCTT GAACTCATGGCTCATATTG	AB 502 bp; CD 384 bp; For *rpfC*$^{\Delta TM1\text{-}2}$, Δ*rpfF-rpfC*$^{\Delta TM1\text{-}2}$ construction
**ΔTM23**	A: GCGAATTC CATGGCCACGCTCAGCTTC<br>B: GCCATATG CAAGGTATCGCCGTGGGTG<br>C: GCCATATG GGCAACGGTCTGCGCTATG<br>D: GCAAGCTT CACCTCTTCCACCAGCGAC	AB 380 bp; CD 387 bp; For *rpfC*$^{\Delta TM2\text{-}3}$, Δ*rpfF-rpfC*$^{\Delta TM2\text{-}3}$ construction
**ΔTM34**	A: GCGAATTC CTTCGCAGCACGGAAGGATC<br>B: GCCATATG GGTGCAGGTGTAGTCGAG<br>C: GCCATATG AAAGCCAATCCCTATCTG<br>D: GCAAGCTT CACCTCTTCCACCAGCGAC	AB 333 bp; CD 291 bp; For *rpfC*$^{\Delta TM3\text{-}4}$, Δ*rpfF-rpfC*$^{\Delta TM3\text{-}4}$ construction
**ΔTM45**	A: GCGAATTC AATCTGATCCGCATCATC<br>B: GCCATATG GTTGCTGCCATAGCGCAG<br>C: GCCATATG TTACGTGCGATGACCCGTG	AB 306 bp; CD 315 bp; For *rpfC*$^{\Delta TM4\text{-}5}$, Δ*rpfF-rpfC*$^{\Delta TM4\text{-}5}$

	D: GCAAGCTT CTGCAGGATCAAGTTGAC	construction
**Δinput**	A: GCGAATTC GTGCTGATCTTTTGCCGG<br>B: GCCATATG CATAGACGCCTCAGACGCGC<br>C: GCCATATG TTACGTGCGATGACCCGTG<br>D: GCAAGCTT CTGCAGGATCAAGTTGAC	AB 439 bp; CD 315 bp; For *rpfC*$^{\Delta input}$, Δ*rpfF*-*rpfC*$^{\Delta input}$ construction
**Pro-RpfCΔinput**	A:CGCATATGTTACGTGCGATGACCCGTG<br>B: CGAAGCTTTTCGTTGCTACGGGGGGAG	1668 bp; for protein RpfC$^{\Delta input}$ expression
**Pro-RpfCFL**	A: CATATGAAGTCTCCATTGCCATGG<br>B: AAGCTTTTCGTTGCTACGGGGGGA	2181 bp; for protein RpfC expression
**Pro-H198A**	A:CTTCCTGGCCAATATGAGCGCTGAGTTCCGCACG<br>B:AGCGCTCATATTGGCCAGGAAGCGGCTCTTGGCC	For the H198A point mutated protein of RpfC expression
**Pro-D512V**	A:GACGCGGTGATTGTGGTCCTGCACATGCCGG<br>B: GACCACAATCACCGCGTCGTAATCCTCTTC	For the D512V point mutated protein of RpfC expression
**Pro-H657A**	A:CAACTGCGCGAGAGTGCGGCTGCCTTACGCGGC<br>B: AGCCGCACTCTCGCGCAGTTGTTCCCAGTCCGA	For the H657A point mutated protein of RpfC expression
**rpfC-K2A**	A: CGATTGCAAGCTTATGGCGTCTCCATTGC<br>B: GCCATAAGCTTGCAATCGAATTCCCGCG	For the recombinant vector pHM1:: *rpfC*（K2A） construction
**rpfC-S3A**	A: GATTGCAAGCTTATGAAGGCTCCATTGCC<br>B: CCTTCATAAGCTTGCAATCGAATTCCCGC	For the recombinant vector pHM1:: *rpfC*(S3A) construction
**rpfC-P4A**	A: GATTGCAAGCTTATGAAGTCTGCATTGCCATGG<br>B: CAGACTTCATAAGCTTGCAATCGAATTCCCGCG	For the recombinant vector pHM1:: *rpfC*(P4A) construction
**rpfC-L5A**	A: CAAGCTTATGAAGTCTCCAGCGCCATGGTTG<br>B: GCTGGAGACTTCATAAGCTTGCAATCGAATTC	For the recombinant vector pHM1:: *rpfC*(L5A) construction
**rpfC-P6A**	A: GCTTATGAAGTCTCCATTGGCATGGTTGAAG<br>B: CCAATGGAGACTTCATAAGCTTGCAATCGA	For the recombinant vector pHM1:: *rpfC*(P6A) construction
**rpfC-W7A**	A: TGAAGTCTCCATTGCCAGCGTTGAAGCGG<br>B: GCTGGCAATGGAGACTTCATAAGCTTGCA	For the recombinant vector pHM1:: *rpfC*(W7A) construction
**rpfC-L8A**	A: GTCTCCATTGCCATGGGCGAAGCGGCGC<br>B: GCCCATGGCAATGGAGACTTCATAAGCTTG	For the recombinant vector pHM1:: *rpfC*(L8A) construction

**rpfC-K9A**	A: CTCCATTGCCATGGTTGGCGCGGCGCCTG<br>B: GCCAACCATGGCAATGGAGACTTCATAAG	For the recombinant vector pHM1:: *rpfC*(K9A) construction
**rpfC-R10A**	A: CATTGCCATGGTTGAAGGCGCGCCTGTCAG<br>B: GCCTTCAACCATGGCAATGGAGACTTCAT	For the recombinant vector pHM1:: *rpfC*(R10A) construction
**rpfC-R11A**	A: GCCATGGTTGAAGCGGGCCCTGTCAGGG<br>B: GCCCGCTTCAACCATGGCAATGGAGACT	For the recombinant vector pHM1:: *rpfC*(R11A) construction
**rpfC-L12A**	A: CATGGTTGAAGCGGCGCGCGTCAGGGCGAG<br>B: GCGCGCCGCTTCAACCATGGCAATGGAGAC	For the recombinant vector pHM1:: *rpfC*(L12A) construction
**rpfC-S13A**	A:GGTTGAAGCGGCGCCTGGCAGGGCGAGC<br>B: CCAGGCGCCGCTTCAACCATGGCAATGG	For the recombinant vector pHM1:: *rpfC*(S13A) construction
**rpfC-G14A**	A: GAAGCGGCGCCTGTCAGCGCGAGCAGATTC<br>B: GCTGACAGGCGCCGCTTCAACCATGGCA	For the recombinant vector pHM1:: *rpfC*(G14A) construction
**rpfC-R15A**	A: GCGGCGCCTGTCAGGGGCAGCAGATTCG<br>B: GCCCCTGACAGGCGCCGCTTCAACCATG	For the recombinant vector pHM1:: *rpfC*(R15A) construction
**rpfC-A16D**	A: GCGCCTGTCAGGGCGAGACGATTCGGAAC<br>B: GTCTCGCCCTGACAGGCGCCGCTTCAACC	For the recombinant vector pHM1:: *rpfC*(A16D) construction
**rpfC-D17A**	A: CCTGTCAGGGCGAGCAGCTTCGGAACAC<br>B: GCTGCTCGCCCTGACAGGCGCCGCTTCAAC	For the recombinant vector pHM1:: *rpfC*(D17A) construction
**rpfC-S18A**	A: CTGTCAGGGCGAGCAGATGCGGAACACGC<br>B: CATCTGCTCGCCCTGACAGGCGCCGCTTC	For the recombinant vector pHM1:: *rpfC*(S18A) construction
**rpfC-E19A**	A: CAGGGCGAGCAGATTCGGCACACGCGCAG<br>B: GCCGAATCTGCTCGCCCTGACAGGCGCCG	For the recombinant vector pHM1:: *rpfC*(E19A) construction
**rpfC-H20A**	A: GCGAGCAGATTCGGAAGCCGCGCAGAATC	For the recombinant

	B: GCTTCCGAATCTGCTCGCCCTGACAGGC	vector pHM1:: *rpfC*(H20A) construction
**rpfC-A21D**	A: GAGCAGATTCGGAACACGACCAGAATCTGA<br>B: GTCGTGTTCCGAATCTGCTCGCCCTGACAG	For the recombinant vector pHM1:: *rpfC*(A21D) construction
**rpfC-Q22A**	A: AGATTCGGAACACGCGGCGAATCTGATC<br>B: GCCGCGTGTTCCGAATCTGCTCGCCCTG	For the recombinant vector pHM1:: *rpfC*(Q22A) construction
**Pro-S3A**	A: TTAAGAAGGAGATATACATATGATGAAGG<br>B: GGCAATGGAGCCTTCATCATATGTATATC	For the S3A point mutated protein of RpfC expression
**rpfC-R15K**	A: AAGCGGCGCCTGTCAGGGAAAGCAGATTCG<br>B: TTCCCTGACAGGCGCCGCTTCAACCATGGC	For the recombinant vector pHM1:: *rpfC*(R15K) construction
**rpfC-R15H**	A: AGCGGCGCCTGTCAGGGCATGCAGATTCGG<br>B: ATGCCCTGACAGGCGCCGCTTCAACCATGG	For the recombinant vector pHM1:: *rpfC*(R15H) construction
**rpfC-A16V**	A: GGCGCCTGTCAGGGCGAGTAGATTCGGAA<br>B: ACTCGCCCTGACAGGCGCCGCTTCAACCA	For the recombinant vector pHM1:: *rpfC*(A16V) construction
**rpfC-A16G**	A: GGCGCCTGTCAGGGCGAGGAGATTCGGAAC<br>B: CCTCGCCCTGACAGGCGCCGCTTCAACCA	For the recombinant vector pHM1:: *rpfC*(A16G) construction
**rpfC-D17E**	A: GCCTGTCAGGGCGAGCAGAATCGGAACACG<br>B: TTCTGCTCGCCCTGACAGGCGCCGCTTCAAC	For the recombinant vector pHM1:: *rpfC*(D17E) construction
**rpfC-D17N**	A: CGCCTGTCAGGGCGAGCAAATTCGGAACAC<br>B: TTGCTCGCCCTGACAGGCGCCGCTTCAAC	For the recombinant vector pHM1:: *rpfC*(D17N) construction
**rpfC-E19D**	A: GGGCGAGCAGATTCGGACCACGCGCAGA<br>B: GTCCGAATCTGCTCGCCCTGACAGGCGC	For the recombinant vector pHM1:: *rpfC*(E19D) construction
**rpfC-E19Q**	A: TCAGGGCGAGCAGATTCGCAACACGCGCAG<br>B: GCGAATCTGCTCGCCCTGACAGGCGCCGC	For the recombinant vector pHM1::

		rpfC(E19Q) construction
**rpfC-A21V**	A: GAGCAGATTCGGAACACGTCCAGAATCTGA<br>B: GACGTGTTCCGAATCTGCTCGCCCTGACAG	For the recombinant vector pHM1:: *rpfC*(A21V) construction
**rpfC-A21G**	A: GAGCAGATTCGGAACACGGGCAGAATCTG<br>B: CCGTGTTCCGAATCTGCTCGCCCTGACAG	For the recombinant vector pHM1:: *rpfC*(A21G) construction
**rpfC-Q22N**	A: GCAGATTCGGAACACGCGAACAATCTGATCC<br>B: GTTCGCGTGTTCCGAATCTGCTCGCCCTGAC	For the recombinant vector pHM1:: *rpfC*(Q22N) construction
**rpfC-Q22E**	A: GCAGATTCGGAACACGCGGAGAATCTGATC<br>B: CCGCGTGTTCCGAATCTGCTCGCCCTGAC	For the recombinant vector pHM1:: *rpfC*(Q22E) construction
**rpfC-S18N**	A: CTGTCAGGGCGAGCAGATAACGAACACGCGC<br>B: GTTATCTGCTCGCCCTGACAGGCGCCGCTTC	For the recombinant vector pHM1:: *rpfC*(S18N) construction
**rpfC-S18Q**	A: CTGTCAGGGCGAGCAGATCAAGAACACGCGC<br>B: TTGATCTGCTCGCCCTGACAGGCGCCGCTTC	For the recombinant vector pHM1:: *rpfC*(S18Q) construction
**rpfC-S18D**	A: CTGTCAGGGCGAGCAGATGACGAACACGCGC<br>B: GTCATCTGCTCGCCCTGACAGGCGCCGCTTC	For the recombinant vector pHM1:: *rpfC*(S18D) construction
**rpfC-S18E**	A: CTGTCAGGGCGAGCAGATGAAGAACACGCGC<br>B: TTCATCTGCTCGCCCTGACAGGCGCCGCTTC	For the recombinant vector pHM1:: *rpfC*(S18E) construction
**rpfC-S18K**	A: CTGTCAGGGCGAGCAGATAAAGAACACGCGC<br>B: TTTATCTGCTCGCCCTGACAGGCGCCGCTTC	For the recombinant vector pHM1:: *rpfC*(S18K) construction
**rpfC-S18R**	A: CTGTCAGGGCGAGCAGATCGCGAACACGCGC<br>B: GCGATCTGCTCGCCCTGACAGGCGCCGCTTC	For the recombinant vector pHM1:: *rpfC*(S18R) construction
**rpfC-S18H**	A: CTGTCAGGGCGAGCAGATCACGAACACGCGC<br>B: GTGATCTGCTCGCCCTGACAGGCGCCGCTTC	For the recombinant vector pHM1:: *rpfC*(S18H)

		construction
**rpfC-S18G**	A: CTGTCAGGGCGAGCAGATGGGGAACACGCG<br>B: CCATCTGCTCGCCCTGACAGGCGCCGCTTC	For the recombinant vector pHM1:: *rpfC*(S18G) construction
**rpfC-S18V**	A: CTGTCAGGGCGAGCAGATGTCGAACACGCGC<br>B: GACATCTGCTCGCCCTGACAGGCGCCGCTTC	For the recombinant vector pHM1:: *rpfC*(S18V) construction
**rpfC-S18L**	A: CTGTCAGGGCGAGCAGATCTCGAACACGCGC<br>B: GAGATCTGCTCGCCCTGACAGGCGCCGCTTC	For the recombinant vector pHM1:: *rpfC*(S18L) construction
**rpfC-S18I**	A: CTGTCAGGGCGAGCAGATATCGAACACGCGC<br>B: GATATCTGCTCGCCCTGACAGGCGCCGCTTC	For the recombinant vector pHM1:: *rpfC*(S18I) construction
**rpfC-S18P**	A: CTGTCAGGGCGAGCAGATCCCGAACACGCGC<br>B: GGGATCTGCTCGCCCTGACAGGCGCCGCTTC	For the recombinant vector pHM1:: *rpfC*(S18P) construction
**rpfC-S18F**	A: CTGTCAGGGCGAGCAGATTTCGAACACGCGC<br>B: GAAATCTGCTCGCCCTGACAGGCGCCGCTTC	For the recombinant vector pHM1:: *rpfC*(S18F) construction
**rpfC-S18Y**	A: CTGTCAGGGCGAGCAGATTACGAACACGCGC<br>B: GTAATCTGCTCGCCCTGACAGGCGCCGCTTC	For the recombinant vector pHM1:: *rpfC*(S18Y) construction
**rpfC-S18W**	A: CTGTCAGGGCGAGCAGATTGGGAACACGCGC<br>B: CCAATCTGCTCGCCCTGACAGGCGCCGCTTC	For the recombinant vector pHM1:: *rpfC*(S18W) construction
**rpfC-S18C**	A: CTGTCAGGGCGAGCAGATTGCGAACACGCGC<br>B: GCAATCTGCTCGCCCTGACAGGCGCCGCTTC	For the recombinant vector pHM1:: *rpfC*(S18C) construction
**rpfC-S18M**	A: CTGTCAGGGCGAGCAGATATGGAACACGCGC<br>B: CATATCTGCTCGCCCTGACAGGCGCCGCTTC	For the recombinant vector pHM1:: *rpfC*(S18M) construction
**rpfC-S18T**	A: CTGTCAGGGCGAGCAGATACGGAACACGC<br>B: TATCTGCTCGCCCTGACAGGCGCCGCTTC	For the recombinant vector pHM1:: *rpfC*(S18T) construction

**rpfC-L172A**	A: CTGTACTTCGACTCGCTGGCACGTGCGATG<br>B: GCCAGCGAGTCGAAGTACAGCGGCACCGCA	For the recombinant vector pHM1:: *rpfC*(L172A) construction
**rpfC-R173A**	A: TACTTCGACTCGCTGTTAGCTGCGATGACC<br>B: CGTAACAGCGAGTCGAAGTACAGCGGCACC	For the recombinant vector pHM1:: *rpfC*(R173A) construction
**rpfC-A174D**	A: TCGACTCGCTGTTACGTGACATGACCCGTG<br>B: GTCACGTAACAGCGAGTCGAAGTACAGCGG	For the recombinant vector pHM1:: *rpfC*(A174D) construction
**rpfC-M175A**	A: GACTCGCTGTTACGTGCGGCGACCCGTGCA<br>B: GCCGCACGTAACAGCGAGTCGAAGTACAGC	For the recombinant vector pHM1:: *rpfC*(M175A) construction
**rpfC-T176A**	A: TCGCTGTTACGTGCGATGGCCCGTGCAGT<br>B: CCATCGCACGTAACAGCGAGTCGAAGTAC	For the recombinant vector pHM1:: *rpfC*(T176A) construction
**rpfC-R177A**	A: CTGTTACGTGCGATGACCGCTGCAGTGCGC<br>B: GCGGTCATCGCACGTAACAGCGAGTCGAAG	For the recombinant vector pHM1:: *rpfC*(R177A) construction
**rpfC-A178D**	A: TACGTGCGATGACCCGTGACGTGCGCGAAG<br>B: GTCACGGGTCATCGCACGTAACAGCGAGTC	For the recombinant vector pHM1:: *rpfC*(A178D) construction
**rpfC-V179A**	A: GTGCGATGACCCGTGCAGCGCGCGAAGCC<br>B: GCTGCACGGGTCATCGCACGTAACAGCGA	For the recombinant vector pHM1:: *rpfC*(V179A) construction
**rpfC-R180A**	A: GCGATGACCCGTGCAGTGGCCGAAGCCCGG<br>B: GCCACTGCACGGGTCATCGCACGTAACAGC	For the recombinant vector pHM1:: *rpfC*(R180A) construction
**rpfC-E181A**	A: TGACCCGTGCAGTGCGCGCAGCCCGGCAC<br>B: GCGCGCACTGCACGGGTCATCGCACGTAA	For the recombinant vector pHM1:: *rpfC*(E181A) construction
**rpfC-A182D**	A: CCCGTGCAGTGCGCGAAGACCGGCACGCC<br>B: TCTTCGCGCACTGCACGGGTCATCGCACG	For the recombinant vector pHM1:: *rpfC*(A182D) construction
**rpfC-R183A**	A: CGTGCAGTGCGCGAAGCCGCGCACGCCAAC	For the recombinant

	B: GCGGCTTCGCGCACTGCACGGGTCATCGCA	vector pHM1:: *rpfC*(R183A) construction
**rpfC-H184A**	A: GCAGTGCGCGAAGCCCGGGCCGCCAACCAG<br>B: GCCCGGGCTTCGCGCACTGCACGGGTCATC	For the recombinant vector pHM1:: *rpfC*(H184A) construction
**rpfC-A185D**	A: TGCGCGAAGCCCGGCACGACAACCAGGCC<br>B: TCGTGCCGGGCTTCGCGCACTGCACGGGT	For the recombinant vector pHM1:: *rpfC*(A185D) construction
**rpfC-N186A**	A: CGCGAAGCCCGGCACGCCGCCCAGGCCAAG<br>B: GCGGCGTGCCGGGCTTCGCGCACTGCACGG	For the recombinant vector pHM1:: *rpfC*(N186A) construction
**rpfC-Q187A**	A: GAAGCCCGGCACGCCAACGCGGCCAAGAGC<br>B: GCGTTGGCGTGCCGGGCTTCGCGCACTGCA	For the recombinant vector pHM1:: *rpfC*(Q187A) construction
**Pro-RpfCΔsensor**	A: GCCATATGAATCTGATCCGCATCATCATCACC<br>B: GCGTCGACTTCGTTGCTACGGGGGGAG	2115 bp; for protein $RpfC_{\Delta}$sensor expression
**Pro-Sensor-GST**	A: GCGGATCCATGAAGTCTCCATTGCC<br>B: GCGTCGACTCACTGCGCGTGTTCCGAATC	69 bp; for protein Sensor-GST expression
**Pro-RpfGFL**	A: CATATGCAGGATGTTTTAGGGAAT<br>B: AAGCTTCACCCCAGGACGCGCCGA	1137 bp; for protein RpfG expression
**Pro-RpfGD80V**	A: GGATCTGTTGCTGCTCGTGTACCGGATGCC<br>B: CACGAGCAGCAACAGATCCACGCGACCAGCT	For the D80V point mutated protein of RpfG expression
